# Nanobody-mediated neutralization of candidalysin prevents epithelial damage and inflammatory responses that drive vulvovaginal candidiasis pathogenesis

**DOI:** 10.1128/mbio.03409-23

**Published:** 2024-02-13

**Authors:** Marisa Valentine, Paul Rudolph, Axel Dietschmann, Antzela Tsavou, Selene Mogavero, Sejeong Lee, Emily L. Priest, Gaukhar Zhurgenbayeva, Nadja Jablonowski, Sandra Timme, Christian Eggeling, Stefanie Allert, Edward Dolk, Julian R. Naglik, Marc T. Figge, Mark S. Gresnigt, Bernhard Hube

**Affiliations:** 1 Department of Microbial Pathogenicity Mechanisms, Leibniz Institute for Natural Product Research and Infection Biology–Hans Knöll Institute, Jena, Germany; 2 Applied Systems Biology, Leibniz Institute for Natural Product Research and Infection Biology-Hans Knöll Institute, Jena, Germany; 3 Faculty of Biological Sciences, Friedrich Schiller University, Jena, Germany; 4 Junior Research Group Adaptive Pathogenicity Strategies, Leibniz Institute for Natural Product Research and Infection Biology–Hans Knöll Institute, Jena, Germany; 5 Centre for Host-Microbiome Interactions, Faculty of Dentistry, Oral and Craniofacial Sciences, King’s College London, London, England, United Kingdom; 6 Institute of Applied Optics and Biophysics, Friedrich Schiller University, Jena, Germany; 7 Cluster of Excellence Balance of the Microverse, Friedrich Schiller University, Jena, Germany; 8 Biophysical Imaging, Leibniz Institute of Photonic Technology, Jena, Germany; 9 Jena Center for Soft Matter (JCSM), Jena, Germany; 10 QVQ B.V, Utrecht, The Netherlands; 11 Institute of Microbiology, Friedrich-Schiller-University, Jena, Germany; The University of British Columbia, Vancouver, British Columbia, Canada

**Keywords:** candidalysin, vulvovaginal candidiasis, inflammation, cytotoxicity, therapeutic strategy

## Abstract

*Candida albicans* can cause mucosal infections in humans. This includes oropharyngeal candidiasis, which is commonly observed in human immunodeficiency virus infected patients, and vulvovaginal candidiasis (VVC), which is the most frequent manifestation of candidiasis. Epithelial cell invasion by *C. albicans* hyphae is accompanied by the secretion of candidalysin, a peptide toxin that causes epithelial cell cytotoxicity. During vaginal infections, candidalysin-driven tissue damage triggers epithelial signaling pathways, leading to hyperinflammatory responses and immunopathology, a hallmark of VVC. Therefore, we proposed blocking candidalysin activity using nanobodies to reduce epithelial damage and inflammation as a therapeutic strategy for VVC. Anti-candidalysin nanobodies were confirmed to localize around epithelial-invading *C. albicans* hyphae, even within the invasion pocket where candidalysin is secreted. The nanobodies reduced candidalysin-induced damage to epithelial cells and downstream proinflammatory responses. Accordingly, the nanobodies also decreased neutrophil activation and recruitment. *In silico* mathematical modeling enabled the quantification of epithelial damage caused by candidalysin under various nanobody dosing strategies. Thus, nanobody-mediated neutralization of candidalysin offers a novel therapeutic approach to block immunopathogenic events during VVC and alleviate symptoms.

**IMPORTANCE:**

Worldwide, vaginal infections caused by *Candida albicans* (VVC) annually affect millions of women, with symptoms significantly impacting quality of life. Current treatments are based on anti-fungals and probiotics that target the fungus. However, in some cases, infections are recurrent, called recurrent VVC, which often fails to respond to treatment. Vaginal mucosal tissue damage caused by the *C. albicans* peptide toxin candidalysin is a key driver in the induction of hyperinflammatory responses that fail to clear the infection and contribute to immunopathology and disease severity. In this pre-clinical evaluation, we show that nanobody-mediated candidalysin neutralization reduces tissue damage and thereby limits inflammation. Implementation of candidalysin-neutralizing nanobodies may prove an attractive strategy to alleviate symptoms in complicated VVC cases.

## INTRODUCTION

The yeast *Candida albicans* is normally a harmless commensal that colonizes mucosae of the gastrointestinal tract, oral cavity, and vagina (1–3). Under pre-disposing conditions, *C. albicans* can cause mucosal infections that severely impact quality of life (4). Oropharyngeal candidiasis (OPC) is the predominant opportunistic oral infection in individuals infected with human immunodeficiency virus (HIV) and is indicative of HIV disease (5). Vulvovaginal candidiasis (VVC) affects 75% of women at least once during their reproductive years, and more than 5% of women are diagnosed with recurrent vulvovaginal candidiasis (RVVC), having four or more infections annually (6, 7). Alarmingly, this translates to about 138 million women with RVVC per year globally (8). While VVC is associated with microbial dysbiosis, high estrogen levels, behavioral practices, and uncontrolled diabetes mellitus, an immunocompromised immune status rarely pre-disposes women to VVC (6).

*C. albicans* has several virulence factors including adhesins, invasins, hydrolases, and the ability to transition between a yeast and hyphal morphology (4, 9). However, epithelial inflammatory and repair responses, as well as mucosal damage and necrotic cell death, are predominantly triggered by the peptide toxin candidalysin (10–15). Prior to secretion, candidalysin is embedded into a polyprotein precursor, Ece1, which consists of a secretion signal peptide, the precursor peptide for candidalysin, and seven other Ece1 peptides. This structure is likely required to prevent autoaggregation owing to the amphipathic and hydrophobic features of the candidalysin peptide (16). In fact, synthetic candidalysin spontaneously forms aggregates in aqueous solution (17).

In oral epithelial cells (OECs), candidalysin-mediated activation of epithelial growth factor receptor (EGFR) induces mitogen-activated protein kinase (MAPK) signaling, resulting in c-Fos transcription factor and MAPK phosphatase-1 activation (18, 19). This results in the release of inflammatory cytokines and activation of potent innate immune responses (18–20). The epithelial response against *C. albicans* is further augmented by the candidalysin-triggered release of alarmins, anti-microbial peptides, and damage-associated molecular patterns that drive immune cell recruitment (21).

In contrast to OPC, the candidalysin-induced immune response during VVC is not protective (22, 23). While neutrophils are recruited in large numbers, they do not promote fungal clearance (24). This dysfunctionality has been attributed to specific host factors in the vaginal environment, including heparan sulfate, anti-*C*. *albicans* antibodies, and perinuclear anti-neutrophil cytoplasmic antibodies (25, 26).

VVC can be prevented or treated using probiotics and/or azoles (6, 22). Nevertheless, VVC is not always cured, and treatment can be complicated by anti-fungal resistance. Women often experience recurring infections even after anti-fungal treatment (7). Treatment of RVVC requires maintenance-suppressive azole therapy (6). Therefore, unlike in OPC where candidalysin induces a protective anti-fungal immune response (27), the neutralization of candidalysin or modulation of downstream inflammatory responses has been suggested as a therapeutic strategy to prevent immunopathology and symptomology during VVC (10, 28).

Given the crucial role of candidalysin in causing epithelial damage and driving inflammatory responses that underlie VVC pathogenesis, we combined *in vitro* infection models with *in silico* modeling to explore nanobody-mediated neutralization as a potential therapeutic strategy to prevent epithelial damage and inflammatory cytokine release.

## RESULTS

### A llama-derived, candidalysin-neutralizing nanobody

We recently described two V_H_H nanobody clones that exhibited high affinity toward candidalysin: CAL1-F1 and CAL1-H1 (29). We reasoned that binding of the nanobodies may neutralize the biological activity of the toxin and thereby prevent host cell lysis. To assess whether these nanobodies could neutralize candidalysin cytotoxicity, we first used a well-characterized *in vitro* oral epithelial model with which the mechanisms underlying candidalysin-induced cytotoxicity and immune responses were discovered (11).

**Fig 1:**
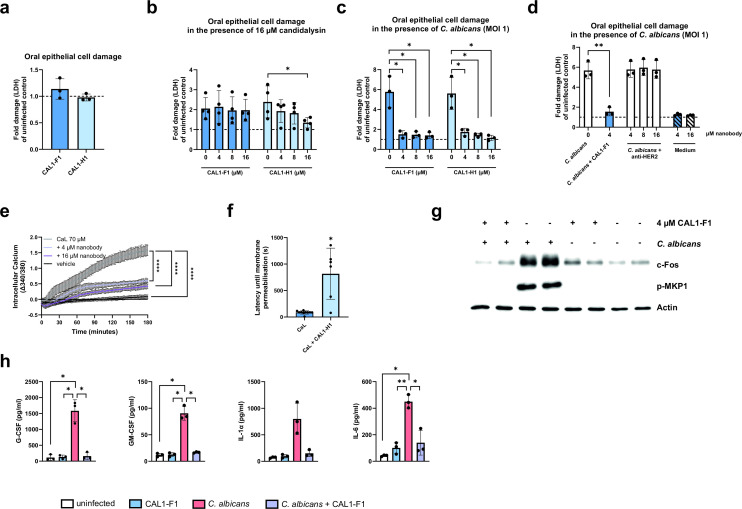
Anti-candidalysin nanobodies neutralize candidalysin-mediated *C. albicans* damage, epithelial signaling, and cytokine responses in TR146 oral epithelial cells (OECs). (**a**) OEC damage after 24 h in the presence of 16 µM anti-candidalysin nanobodies, measured by quantifying lactate dehydrogenase (LDH) activity in the supernatant and presented as fold change of uninfected control (dotted line). OEC damage after 24 h induced by (**b**) 16 µM candidalysin and (**c**) *C. albicans* (multiplicity of infection [MOI] 1) in the presence of increasing concentrations (4, 8, and 16 µM) of anti-candidalysin nanobody. Nanobodies were pre-incubated for 1 h with candidalysin or *C. albicans* before addition to OECs. Damage was measured by quantifying LDH activity in the supernatant and presented as fold change of uninfected control (dotted line). (**d**) OEC damage after 24 h induced by *C. albicans* (MOI 1) in the presence of increasing concentrations (4, 8, and 16 µM) of a control V_H_H nanobody, anti-human epidermal growth factor receptor 2 (anti-HER2), compared to 4 µM of anti-candidalysin nanobody CAL1-F1. Nanobodies were pre-incubated for 1 h with *C. albicans* before addition to OECs. Damage was measured by quantifying LDH activity in the supernatant and presented as fold change of uninfected control (dotted line). (**e**) Calcium influx into OECs measured over 3 h after treatment with 70 µM candidalysin (CaL) pre-incubated with and without anti-candidalysin nanobody (4 and 16 µM). (f) Latencies until lipid bilayer permeability were measured after treatment with 10 µM candidalysin in the absence and presence of 10 µM nanobody. (**g**) c-Fos and p-MKP1 induction in OECs 2 h after infection with *C. albicans* (MOI 0.01) that was pre-incubated with and without anti-candidalysin nanobody CAL1-F1. Image is representative of *n* = 3. (**h**) Granulocyte colony-stimulating factor (G-CSF), granulocyte-macrophage colony-stimulating factor (GM-CSF), interleukin (IL)-1α, and IL-6 released by OECs 24 h after infection with *C. albicans* (MOI 0.01) pre-incubated with and without 4 µM anti-candidalysin nanobody CAL1-F1. Bars represent the mean ± standard deviation of *n* = 3 (**a, c, d, e, and h**), *n* = 4 (b), or *n* = 6 (f) independent replicates. Means were compared for significance using paired *t*-tests (**b–d**), Kruskal-Wallis test with a Dunn multiple comparisons test compared to the candidalysin control (**e**), Mann-Whitney test (**f**), and one-way analysis of variance with a Dunnett multiple comparisons test compared to the *C. albicans*-infected control (**h**). Statistical significance is indicated as *P ≤ 0.05, **P ≤ 0.01, and ****P ≤ 0.0001.

Potential detrimental effects of nanobodies to OECs were excluded as even the highest concentration (16 µM) of nanobody did not cause cytotoxicity of OECs (Fig. 1a). While only the CAL1-H1 nanobody reduced synthetic candidalysin-induced damage (Fig. 1b), both nanobody clones abolished OEC damage caused by wild-type *C. albicans* (Fig. 1c). To verify that this neutralization effect is due to the candidalysin-specific activity of the V_H_H nanobodies, we tested a V_H_H nanobody that does not bind candidalysin. Unlike the anti-candidalysin V_H_H nanobody CAL1-F1, the control V_H_H nanobody failed to reduce *C. albicans*-induced damage of OECs (Fig. 1d).

Protection against epithelial cell death was likely associated with neutralization of the cytolytic pore-forming capacity of candidalysin as the nanobody prevented calcium influx into OECs following exposure to synthetic candidalysin (Fig. 1e). Accordingly, the anti-candidalysin nanobody also significantly delayed the capacity of synthetic candidalysin to compromise membrane integrity (Fig. 1f). Furthermore, levels of c-Fos and phosphorylated MKP1 were reduced following *C. albicans* infection in the presence of nanobodies (Fig. 1g), suggesting minimal epithelial activation. Consequently, no release of the proinflammatory mediators granulocyte colony-stimulating factor (G-CSF), granulocyte-macrophage colony-stimulating factor (GM-CSF), interleukin (IL)-1α, and IL-6 was observed (Fig. 1h). The nanobody did not elicit inflammatory responses.

### Nanobodies reduce candidalysin-induced vaginal epithelium damage

**Fig 2:**
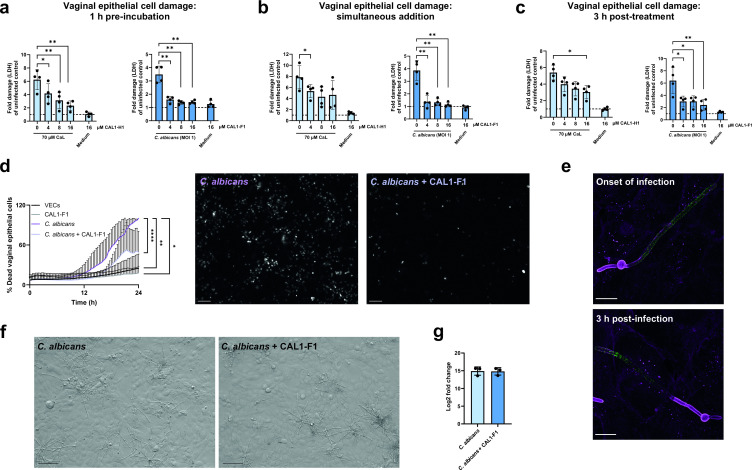
Anti-candidalysin nanobodies reduce candidalysin-induced damage of A-431 vaginal epithelial cells (VECs) without affecting hyphae formation and *ECE1* expression. Damage induced by 70 µM candidalysin or *C. albicans* (MOI 1) on VECs in the presence of increasing concentrations (4, 8, and 16 µM) of anti-candidalysin nanobody. Damage was measured by quantifying lactate dehydrogenase (LDH) activity in the supernatant and presented as fold change of uninfected control (dotted line). Nanobodies were added to VECs (**a**) after pre-incubation with candidalysin or *C. albicans* for 1 h, (**b**) simultaneously with candidalysin or *C. albicans* at the onset of infection, or (**c**) 3 h after VECs were treated with candidalysin or infected with *C. albicans*. (**d**) Percentage of propidium iodide (PI)-positive VECs following infection with *C. albicans* in the absence and presence of 4 µM anti-candidalysin nanobody CAL1-F1 over 24 h. PI staining was used to quantify necrotic cell death dynamics, and the microscopy images are representative of infection in the absence and presence of anti-candidalysin nanobody at 24 h post-infection. Scale bar = 100 µm. (**e**) Immunofluorescence staining of anti-candidalysin nanobody localization (in green) during treatment of *C. albicans*-infected VECs (both in magenta) at the onset and 3 h after infection. Images are representative of *n* = 2 experiments. Scale bar = 10 µm. (**f**) Brightfield microscopic images of *C. albicans* hyphae 6 h after infection of VECs in the absence or presence of anti-candidalysin nanobody. Images are representative of *n* = 2 repeats. Scale bar = 100 µm. (**g**) *ECE1* mRNA expression by *C. albicans* SC5314 24 h after infection of VECs in the absence and presence of 4 µM CAL1-F1 nanobody. Bars represent the mean ± standard deviation of *n* = 4 (**a–c**) or *n* = 3 (**d and g**) independent replicates. Means were compared for significance using paired *t*-tests (**a, b, c, and g**) and Kruskal-Wallis test with a Dunn multiple comparisons test compared to the *C. albicans* control (d). Statistical significance is indicated as *P ≤ 0.05, **P ≤ 0.01, and ****P ≤ 0.0001.

While activation of epithelial signaling pathways during OPC initiates protective neutrophil-mediated responses against *C. albicans* infection (18), epithelial damage and proinflammatory responses are key drivers of VVC pathogenesis (22, 24). Therefore, the therapeutic potential of anti-candidalysin nanobodies for the treatment of VVC was investigated by evaluating their efficacy to neutralize candidalysin-induced vaginal epithelial cell (VEC) damage. Similar to OECs, anti-candidalysin nanobodies alone did not cause VEC cytotoxicity (Fig. 2a through c).

Pre-incubation of 70 µM synthetic candidalysin or *C. albicans* with the CAL-H1 or CAL-F1 anti-candidalysin nanobodies, respectively, significantly reduced VEC damage (Fig. 2a). Simultaneous addition of the nanobody also reduced candidalysin-mediated damage (Fig. 2b). However, for therapeutic purposes, the nanobody should be effective after an infection has been established. Importantly, even delayed addition of the nanobody 3 h after treatment with synthetic toxin or *C. albicans* infection (Fig. 2c) significantly reduced epithelial damage.

The ability of the nanobodies to prevent VEC damage and necrotic cell death was verified using propidium iodide (PI) staining. When candidalysin compromises epithelial cell membrane integrity, PI enters epithelial cells and intercalates within DNA. The number of PI-positive VECs after *C. albicans* infection was significantly reduced by adding anti-candidalysin nanobodies (Fig. 2d), confirming that the nanobodies can prevent *C. albicans*-induced necrotic VEC death.

### Nanobodies localize to invasive *C. albicans* hyphae

Having observed that CAL1-F1 neutralizes epithelial cell damage caused by *C. albicans*, we investigated by immunofluorescence whether the nanobody localizes to the invasion pocket where candidalysin is secreted and causes cytotoxicity (29). Irrespective of whether the nanobody was added at the onset of infection or 3 h after infection, it could be visualized within the invasion pocket formed by invading *C. albicans* hyphae (Fig. 2e).

Addition of the CAL1-F1 nanobody at the onset of infection did not impact *C. albicans* hyphal growth (Fig. 2f) or influence *C. albicans ECE1* mRNA expression during VEC infection (Fig. 2g). Collectively, these data suggest that the anti-candidalysin nanobody prevents VEC damage by neutralizing secreted candidalysin in the invasion pocket.

### Anti-candidalysin nanobodies dampen proinflammatory responses

After observing that anti-candidalysin nanobodies reduce VEC damage, we investigated whether the nanobodies also dampen the proinflammatory responses driving neutrophil recruitment that are known to exacerbate VVC pathogenesis.

**Fig 3:**
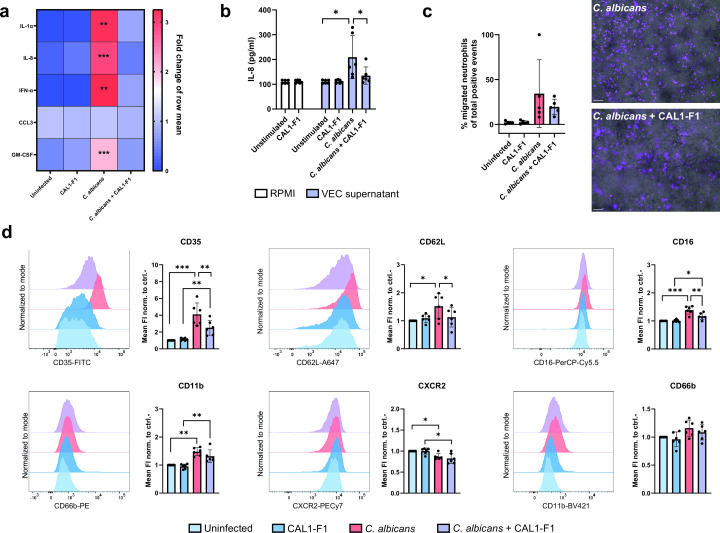
Anti-candidalysin nanobodies dampen proinflammatory responses. (**a**) Heatmap showing fold changes in cytokine release by A-431 vaginal epithelial cells (VECs) in the presence and absence of 4 µM anti-candidalysin nanobodies added 3 h after *C. albicans* infection. Data are presented as fold change of the row mean for each cytokine. (**b**) IL-8 release by primary human neutrophils 24 h after stimulation with supernatants of *C. albicans*-infected VECs in the presence and absence of anti-candidalysin nanobodies. Neutrophils were directly co-incubated with CAL1-F1 nanobodies as a control. (**c**) Representative images (scale bar = 100 µm) and quantified migration of primary human neutrophils toward *C. albicans*-infected VECs in the presence and absence of anti-candidalysin nanobodies as determined by immunofluorescence. Migration was quantified as the percentage of cytopainter green-positive events of the total amount of neutrophils. (**d**) Representative histograms and bar graphs of mean fluorescence intensity (FI) of surface activation markers on neutrophils stimulated with supernatants of *C. albicans*-infected VECs in the presence and absence of anti-candidalysin nanobodies for 3 h. The average mean FI was derived from viable CD15^+^ neutrophils and normalized to unstimulated neutrophils. Bars represent the mean ± standard deviation of *n* = 4 (**a**), *n* = 6 (**b and d**), or *n* = 5 (**c**) independent replicates. Means of the raw data (pg/mL) were compared for significance using one-way analysis of variance (ANOVA) with a Dunnett multiple comparisons test to the uninfected control (**a**). Means were compared using paired *t*-tests (**b**) and two-way ANOVA with a Holm-Šidák multiple comparisons test (**c and d**). Statistical significance is indicated as *P ≤ 0.05, **P ≤ 0.01, and ***P ≤ 0.001.

While we detected IL-1α, IL-8, interferon (IFN)-α, CCL3, and GM-CSF in *C. albicans*-infected VEC supernatants, we did not detect IL-6, IFN-β, CCL2, CCL4, CCL5, CCL20, G-CSF, IL-17, CXCL1, or CXCL2. Nanobodies alone did not induce cytokine secretion by VECs (Fig. 3a; Fig. S1), but they abolished IL-1α, IFN-α, IL-8, and GM-CSF release by *C. albicans*-infected VECs (Fig. 3a). Similarly, there was decreased GM-CSF, IL-1α, and IL-8 release by VECs treated with synthetic candidalysin in the presence of anti-candidalysin nanobodies (Fig. S1). Notably, primary human neutrophils released IL-8 when stimulated with supernatants of infected VECs, but this was abolished in the presence of the anti-candidalysin nanobody (Fig. 3b). Neither the nanobody alone nor supernatants from uninfected VECs treated with the nanobody induced IL-8 release by neutrophils (Fig. 3b). Using a neutrophil chemotaxis assay, we observed reduced neutrophil recruitment to VECs infected in the presence of nanobodies (Fig. 3c). Correspondingly, neutrophil surface expression of CD35, CD62L, CD11b, and CD16 significantly increased, and CXCR2 significantly decreased upon stimulation with supernatants of infected VECs (Fig. 3d), highlighting a state of neutrophil activation. However, when candidalysin-neutralizing nanobodies were present during infection, VEC supernatants induced lower levels of CD35, CD62L, and CD16 surface expression on neutrophils, while there was a trend for reduced CD11b surface expression (Fig. 3d). Similar levels of CXCR2 expression were still observed in the presence of the nanobody. This demonstrates that anti-candidalysin nanobodies can prevent proinflammatory responses during VEC infection, thereby dampening neutrophil activation.

### Modeling candidalysin neutralization *in silico* captures *in vitro* data

Since our data show that anti-candidalysin nanobodies prevent epithelial damage and neutrophil activation, we proposed nanobody-mediated neutralization as a potential therapeutic option to prevent immunopathology during VVC. To investigate possible treatment strategies, we developed an ordinary differential equation model to explore the dynamics of candidalysin neutralization using the anti-candidalysin nanobody (see *In silico* model description in Materials and Methods).

In general, the neutralization model for synthetic candidalysin considers the characteristics of candidalysin and incorporates five features: the anti-candidalysin nanobody (Nb), candidalysin concentration as a monomer $\left(C_{M}\right)$ and aggregate ${(C}_{A})$, cytoplasmic enzyme lactate dehydrogenase (LDH) that is released upon epithelial cell damage, and the proportion of alive VECs (E). The model further integrates the interaction between *C. albicans*-secreted candidalysin and the anti-candidalysin nanobody, when candidalysin forms polymers (30). Three states for *C. albicans* were considered, referring to the amount of yeast (Y), non-invasive filamentous $\left(F_{NI}\right),$ and invasive filamentous $\left(F_{I}\right)$ cells (31).

The parameters of our model were estimated using our *in vitro* data (Fig. 2a through c; Fig. S2). A comparison between the *in vitro* data and the model for the damage caused by candidalysin without nanobody neutralization can be found in Fig. S3. Given the *in vitro* data, parameter $k_{n}$, the degradation rate of the nanobody, is unidentifiable. Thus, we assumed that degradation of the nanobody is negligible within the 24-h time frame of the experimental data (Fig. 2a through c). Other model parameters and their confidence intervals are shown for a candidalysin aggregate size of 8 in Table 1, since candidalysin was previously hypothesized to polymerize in solution to form membrane pores consisting of eight monomers (30). However, as the size of aggregated candidalysin is not known *in vitro* and likely includes much larger aggregates, we screened a range of candidalysin aggregate sizes to assess the sensitivity of the model outcome for different aggregate sizes ranging from 8 to 1,024 monomers. Therefore, error bands across all predictions depict the variability from varying candidalysin aggregate sizes. Furthermore, the parameters $k_{d}$, $k_{s}$, and α have a linear relationship with the aggregate size (Table S1). In addition, we conducted a Sobol sensitivity analysis (32) on the parameters α, $k_{d}$, $k_{a}$, $k_{b}$, and $k_{s}$ with respect to the amount of predicted LDH for the model with synthetic and native candidalysin, each examined with and without anti-candidalysin nanobody at various time points. As can be seen in Fig. S4 and S5, by comparing the first and total order Sobol indices, the influence of the parameters is mostly linear. However, non-linearity becomes more pronounced in the presence of the anti-candidalysin nanobody or when candidalysin is secreted, as in the case of native candidalysin. According to the senstivity analysis, the largest impact on the model outcome is attributed to the parameter controlling the damage caused by candidalysin and the parameter governing the secretion of candidalysin. In contrast, parameters that indirectly influence damage by delaying candidalysin-induced damage through either controlling aggregate formation ($k_{a}$), aggregate depletion (α) or neutralization ($k_{b}$) do not exert a strong influence. Furthermore, the analysis reveals that the sensitivity of all the parameters exhibits a time dependency.

**TABLE 1:** Parameters of the *in silico* candidalysin neutralization model^*a*^

	Parameter	Mean estimate	95% CI	
			Lower	Upper
$k_{b}$	Blocking rate of $C_{M}$ by Nb	7.16	4.3	1.34e+01
$k_{a}$	Transition rate from $C_{M}$ to $C_{A}$	2.33e−05	1.57e−05	3.8e−05
$k_{d}$	Damage rate of $C_{A}$ on E leading to LDH release	3.44	9.27e−01	2.47e+01
$k_{l}$	Degradation rate of LDH	1.64e−06	9.44e−07	2.33e−06
$k_{s}$	Secretion rate for $C_{M}$ by $F_{I}$ for “effective” candidalysin	1.55e−14	9.56e−15	2.23e−14
α	Conversion constant for usage of aggregate on host cell damage	1.87e−05	1.74e−05	2.02e−05
β	Conversion constant for LDH release on host cell death	2.04e+02	1.85e+02	2.26e+02

^*a*^ Identifiable parameters of the model are given for a candidalysin aggregate size of 8 with their confidence intervals.

### *In silico* modeling predicts that nanobodies added 12 h post-infection reduce *C. albicans* damage

**Fig 4:**
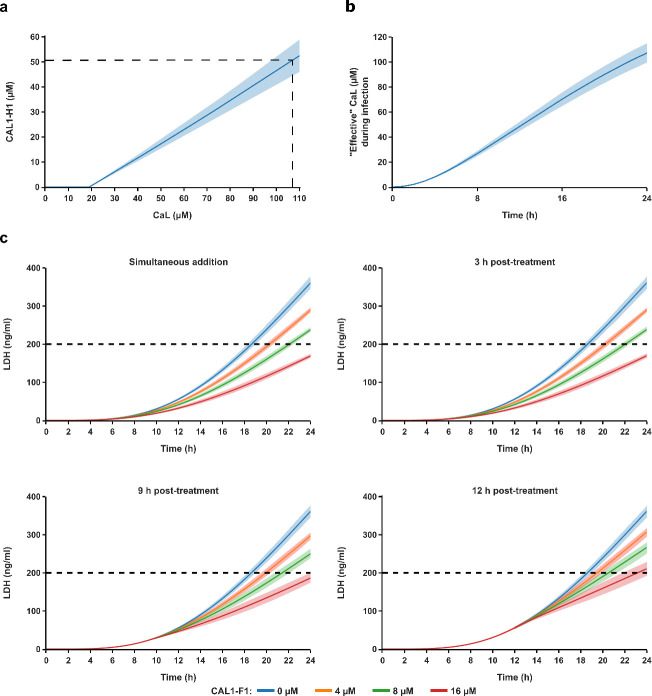
*In silico* modeling of the interaction between anti-candidalysin nanobodies and candidalysin offers insight into optimal treatment strategies. (**a**) Model prediction of the anti-candidalysin nanobody concentration needed to neutralize vaginal epithelial cell (VEC) damage caused by synthetic candidalysin (CaL). Neutralization of cell damage was defined as less than 10% dead of maximum dead VECs [less than 200 ng/mL lactate dehydrogenase (LDH), which corresponds to uninfected VEC controls] in the system after 24 h. (**b**) Model prediction of the cumulative “effective” *C. albicans*-secreted candidalysin that is released over a time span of 24 h. (**c**) Model prediction showing epithelial cell damage as LDH over 24 h when anti-candidalysin nanobodies (0, 4, 8, and 16 µM) were added simultaneously at the onset of *C. albicans* infection or 3–12 h after infection as a post-treatment. Dotted lines indicate the basal level of damage as 200 ng/mL LDH secreted by uninfected VEC controls. Error bands across all plots depict the variability from varying candidalysin aggregate sizes.

The *in silico* model was able to successfully reproduce nanobody-mediated neutralization of epithelial cell damage caused by synthetic candidalysin. The interaction between the anti-candidalysin nanobody and synthetic candidalysin follows a linear pattern, indicating that more nanobody would be required to neutralize epithelial cell damage caused by increasing candidalysin concentrations. Interestingly, neutralization was predicted to occur at ratios in the range of 1:2–1:5 (nanobody:candidalysin), depending on the candidalysin concentration (Fig. 4a). Here, neutralization denotes that less than 10% of the maximum amount of dead VECs in the system is reached at the end of the model simulation. This corresponds to approximately 200 ng/mL of LDH, which represents the basal level of LDH in uninfected VEC controls.

The *in silico* model also allowed us to predict the secretion of “effective” candidalysin by invasive filamentous *C. albicans* cells (Fig. 4b). Since the exact dynamics of candidalysin secretion is unknown, these predictions should be understood as approximations aligning more with synthetic candidalysin rather than with native candidalysin. Therefore, the data represent the equivalent concentration of synthetic candidalysin that is secreted by *C. albicans* over time and is capable of causing epithelial cell damage. Nevertheless, our model allowed us to quantify the cumulative “effective” candidalysin in the system as approximately 107 µM after 24 h (Fig. 4b).

Having shown *in vitro* that the anti-candidalysin nanobody can reduce epithelial cell damage when added 3 h after *C. albicans* infection of VECs (Fig. 2c), we modeled post-treatment to determine up until which time point the nanobody can still be applied to mitigate *C. albicans*-induced VEC damage. We were able to model that nanobody addition at the onset of *C. albicans* infection is more efficient as compared to post-infection treatment (Fig. 4c). When added simultaneously with infection, the nanobody delays the onset of VEC damage (comparing 10% dead VECs in the absence and presence of 8 µM anti-candidalysin nanobody) by approximately 3 h (Fig. 4c). The effectiveness of the nanobody is amplified as the concentration increases. It is predicted that when using 16 µM anti-candidalysin nanobody, epithelial cell damage can be neutralized even when added 9 h after infection. Nevertheless, high nanobody concentrations still reduce epithelial cell damage when added 12 h post-infection.

### Anti-candidalysin nanobody efficacy is comparable to treatment with the anti-fungal fluconazole

**Fig 5:**
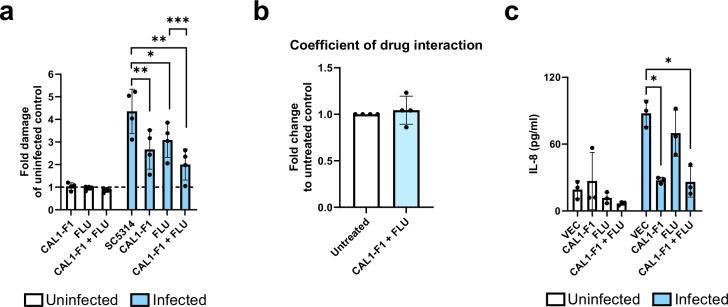
Anti-candidalysin nanobodies reduce *C. albicans-*induced damage of A-431 vaginal epithelial cells (VECs) with similar efficacy as fluconazole. (**a**) VEC damage after 24 h of *C. albicans* infection (MOI 1) with and without 4 µM anti-candidalysin nanobody (CAL1-F1) and/or 4 µg/mL fluconazole (FLU) being added 9 h post-infection. Damage was measured by quantifying LDH activity in the supernatant and presented as fold change of uninfected control (dotted line). (**b**) The combined effect of 4 µg/mL FLU and 4 µM anti-candidalysin nanobody (CAL1-F1) on epithelial cell damage was calculated as the coefficient of drug interaction (CDI) using LDH data. CDI = AB / (A × B), where AB (FLU + nanobody), A (FLU), and B (nanobody) are the fold changes of the untreated infected group. CDI <1, = 1, or >1 indicates synergistic, additive, or antagonistic effects, respectively. (**c**) IL-8 secretion by VECs after 24 h of *C. albicans* infection (MOI 1) with and without anti-candidalysin nanobodies and/or fluconazole being added 9 h after infection. Bars represent the mean ± standard deviation of *n* = 4 (**a and b**) or *n* = 3 (**c**) independent replicates. Means were compared for significance to uninfected and infected controls, as well as between treatments using one-way ANOVA with Tukey and Dunnett multiple comparisons test (**a and c**) and paired *t*-tests (**b**). Statistical significance is indicated as *P < 0.05, **P ≤ 0.01, and ***P ≤ 0.001.

The efficacy of anti-candidalysin nanobodies to prevent VEC damage was compared to fluconazole (FLU), an azole commonly used to treat VVC and a maintenance therapy for RVVC (6). We confirmed our *in silico* model predictions (Fig. 4c) that nanobodies added 9 h post-infection at 4 µM reduce *C. albicans*-induced VEC damage (Fig. 5a). Nanobodies added at this concentration reduced damage of *C. albicans*-infected VECs to a similar extent as 4 µg/mL FLU (Fig. 5a), which is comparable to concentrations in the vaginal niche (6). Moreover, by combining nanobodies with FLU, epithelial cell damage was more effectively reduced than treating with nanobody or FLU alone (Fig. 5a). This was likely due to the neutralization of candidalysin in combination with preventing *C. albicans* growth and thus its capacity to produce more toxin. This effect is additive and not synergistic according to the calculated coefficient of drug interaction (CDI) being close to 1 (Fig. 5b). Interestingly, FLU alone did not show the same efficacy as nanobodies to reduce IL-8 secretion by *C. albicans*-infected VECs (Fig. 5c).

## DISCUSSION

VVC affects millions of women annually, yet treatment options remain limited, and often recurrence is observed. As pathogenesis involves tissue damage and immunopathology that is caused by the *C. albicans* toxin candidalysin (10), we pre-clinically explored nanobody-mediated neutralization of candidalysin as a therapeutic strategy to treat VVC. We observed that a llama-derived anti-candidalysin nanobody dampened epithelial tissue damage caused both by synthetic candidalysin and *C. albicans-*secreted candidalysin during infection *in vitro*. We showed that neutralization of cytotoxicity was associated with reduced activation of OECs. In VECs, neutralization resulted in the reduced release of proinflammatory cytokines and reduced neutrophil activation and recruitment. The data suggest that targeting candidalysin therapeutically could break the hyperinflammatory loop that drives VVC immunopathology and severity of symptoms.

Antibody-mediated neutralization of a microbial toxin has previously been explored for the vaginal pathogen *Gardnerella vaginalis* (33). Antibodies against its cytolytic toxin vaginolysin successfully reduced damage of host cells. Similar findings were observed for nanobodies generated against the Shiga toxin of *Escherichia coli* (34).

Here we show the potential of neutralizing candidalysin as a therapeutic strategy. Anti-candidalysin nanobodies not only neutralized OEC damage during infection but also prevented epithelial activation and downstream cytokine release in infected OECs.

Neutralizing toxins offers an opportunity to block key virulence factors that typically activate the immune system. Thus, toxins are ideal vaccine targets as in the case of tetanus (35, 36). Targeting virulence factors in therapeutics represents a major advantage over traditional anti-microbial therapies as they can be applied without impacting the healthy microbial flora and offer a reduced risk of developing anti-fungal resistance (37, 38). Recently, the *C. albicans*-secreted zinc-binding protein Pra1 was linked to immunopathology and it was shown that RVVC in women can be reduced by inhibiting this fungal factor using zinc treatment (39).

To treat acute VVC infections, topical azoles or oral fluconazole is typically prescribed (6, 40). Fluconazole is also used to treat recurrent and severe infections either as a single dose or maintenance suppressive therapy, yet infections persist in a number of patients irrespective of fluconazole treatment (6, 40–42). However, a drawback of azole therapy is that it also negatively impacts fungi such as *Saccharomyces* species, which are beneficial for the prevention of VVC (43). We show that a candidalysin-neutralizing nanobody exhibits similar efficacy as fluconazole in protecting VECs from cytotoxic damage induced by *C. albicans*. The local fluconazole concentration during treatment is approximately 4–8 µg/mL; thus, our dosage (4 µg/mL) falls within the range of what is expected *in vivo* during infection (6). We also show that the nanobody and fluconazole function additively to reduce VEC damage. Combining anti-candidalysin nanobodies with an anti-fungal drug is an attractive treatment option, as this will reduce fungal growth and hypha formation while concomitantly reducing host cell damage and immunopathology driven by candidalysin.

To further investigate treatment strategies, we developed an *in silico* model to give insight into the interaction between candidalysin and the anti-candidalysin nanobody, which can be used to further explore nanobody application. The nanobody neutralized candidalysin-induced VEC damage in a ratio ranging from 1:2 to 1:5 (nanobody:candidalysin). Based on *in vitro* and *in silico* data, we observed that although more effective when applied at the onset of infection, anti-candidalysin nanobodies can effectively reduce VEC damage when added post-infection. Therefore, the nanobodies are able to neutralize candidalysin in the invasion pocket during an established infection (15, 29). Based on the dynamics of synthetic candidalysin, the amount of “effective” candidalysin that is secreted by *C. albicans* hyphae and capable of VEC damage was predicted to be 107 µM after 24 h. Considering the predicted neutralization ratio, it is recommended that the nanobody should be applied at a maximum daily dose of approximately 50 µM. Our *in vitro* data indicated that the nanobody may exhibit an even increased efficacy *in vivo*, since we observed that nanobodies were more effective at neutralizing damage caused by *C. albicans* (multiplicity of infection [MOI] 1) compared to the addition of synthetic candidalysin to epithelial cells. This phenomenon might be explained by spontaneous aggregation and clumping of the synthetic toxin in aqueous solution (17) and slower and more controlled release of lower concentrations of native candidalysin by *C. albicans* hyphae.

Furthermore, when treating host cells with fungal toxin *in vitro*, we observed that lower nanobody concentrations were effective against 70 µM candidalysin on VECs compared to OECs, where only the highest nanobody concentration reduced epithelial cell damage caused by 16 µM candidalysin, indicating differences, depending on the host cell type. Nevertheless, on both OECs and VECs, even the lowest nanobody concentration was effective at reducing *C. albicans*-induced host cell damage.

VVC is predominantly caused by *C. albicans* (44), the species in which candidalysin was discovered (13, 45). Willems et al. (46) postulated that *C. albicans* is the main etiological agent of VVC, since this species vigorously forms hyphae, expresses candidalysin, and causes immunopathology compared to non-*C. albicans* (NAC) species. Vaginal infections by NAC species are, however, rising and *Candida glabrata* is the second biggest etiological agent of VVC (44). VECs display distinct transcriptional responses to NAC species, whereas the epithelial response to *C. albicans* is primarily driven by candidalysin (12). The only NAC species with known *ECE1* orthologs are *Candida africana*, *Candida dubliniensis*, and *Candida tropicalis* (46–48). However, although *ECE1* gene sequences and peptide structures are relatively conserved between *C. albicans* strains and *Candida* species, the expression pattern of *ECE1* and the biological role of Ece1 for NAC species are unknown (47–49). In *C. albicans* strains, the host cell damage potential of candidalysin is determined by a series of properties including fungal morphology, *ECE1* expression, processing, and secretion (29, 48, 49). The lack of *ECE1*, in addition to morphological differences, further explains why NAC species are generally less pathogenic and do not induce immunopathology as robustly as *C. albicans* (46). Therefore, NAC infections are reported to be less severe, although contrasting findings are reported in literature (44).

Our data show that the nanobodies act directly on candidalysin and prevent the toxin from causing epithelial membrane damage, since we observed less calcium influx and delayed permeabilization of candidalysin-treated OECs and lipid bilayers, respectively, in the presence of nanobodies. This is further supported by microscopy images showing that the anti-candidalysin nanobodies bound candidalysin within the invasion pocket on VECs without affecting hypha formation and *ECE1* expression. Given that blocking downstream effects of candidalysin, such as EGFR signaling, may lead to potentially contradicting disease outcomes (18, 50), directly neutralizing candidalysin seems a far more promising approach. In addition to neutralizing epithelial tissue damage, the anti-candidalysin nanobodies dampened inflammatory responses that drive VVC symptoms. Notably, cytokine secretion by VECs was reduced when nanobodies were added 3 h after *C. albicans* infection. Reduced epithelial damage most likely accounts for lower secretion of the alarmin IL-1α, which leads to reduced IL-8 and GM-CSF release, similar to what has been described for candidalysin-exposed OECs (51).

In line with this, we observed that neutrophils exposed to supernatants of *C. albicans*-infected VECs secreted less IL-8 and showed reduced activation in the presence of nanobodies. CXCR2, an integral receptor for neutrophil migration, showed reduced expression in the presence of *C. albicans* infection. This supported the notion that CXCR2 bound increasing amounts of IL-8 secreted during vaginal infection and was then internalized during migration along the chemokine gradient (52). This effect was, however, not mitigated by the presence of nanobody. Surprisingly, expression of L-selectin (CD62L), a neutrophil marker of adhesion to endothelial cells and migration (53), was increased in response to supernatants of infected VECs and lower in the presence of anti-candidalysin nanobodies. CD62L is expected to be inversely regulated with CD11b during granulocyte activation *in vitro* (54–56), since CD62L is rapidly shed during activation (53). It is, therefore, difficult to determine how the increased CD62L surface expression, which was reduced by the nanobodies, reflects neutrophil activation state. Even though the degranulation marker CD66b was unchanged, CD35, which can be found inside secretory vesicles (57), was increased on the surface by infected VEC supernatants but decreased in the presence of the nanobodies. Overall, these data show that nanobodies can reduce neutrophil activation in response to vaginal epithelial *C. albicans* infection. Comparably, we also showed reduced neutrophil migration during *C. albicans* infection in the presence of anti-candidalysin nanobodies.

Our data show that anti-candidalysin nanobodies may represent a potential strategy to treat VVC by dampening VEC damage and associated inflammatory responses. This, in turn, leads to reduced neutrophil activation and recruitment and thus reduced immunopathology. Collectively, this could result in less severe symptomatic VVC episodes. Nanobodies may prove useful in combination therapy with fluconazole to mitigate the fungal burden, which is not cleared by neutrophils, in parallel with candidalysin-induced inflammation. To successfully implement this therapeutic strategy, future work should prioritize preparing a nanobody formulation that can be applied *in vivo*. Various considerations are needed to develop such a therapy including pharmocokinetic and stability studies (58). Nevertheless, by combining wet bench techniques with bioinformatic modeling, we were able to provide a pre-clinical proof of concept as basis for anti-candidalysin nanobody therapy. Based on our *in vitro* data, we established a mathematical model that can support further development of nanobody-mediated candidalysin neutralization in terms of identifying optimal doses and dosing intervals.

## MATERIALS AND METHODS

### Culture and maintenance of *C. albicans*

*C. albicans* strain SC5314 (59) and BWP17/CIp30 (isogenic to SC5314) were cultured on 1% yeast extract, 2% peptone, and 2% dextrose (YPD) medium (for solid medium, 1.5% agar was supplemented). For infection, a single colony was inoculated into YPD broth that was incubated overnight (ON) at 30°C with shaking at 180 rpm. Yeast cells were washed 3× with phosphate-buffered saline (PBS, pH 7.4). The cell number was enumerated using a Neubauer chamber and adjusted to the number needed for infection.

### Culture of human oral and vaginal epithelial cells

TR146 OECs (ECACC 10032305) and A-431 VECs (DSMZ no. ACC91) were cultured in the presence of 10% heat-inactivated fetal bovine serum (Bio & Sell) in Dulbecco’s modified Eagle medium (DMEM)/F-12 medium (Gibco) and RPMI-1640 medium (Gibco), respectively, according to the supplier’s instructions. Cell lines were authenticated by commercial STR profiling (Eurofins Genomic) and checked for mycoplasma contaminations using a PCR mycoplasma test kit (PromoKine) following the manufacturer’s instructions. For all experiments, unless specified otherwise, cells were seeded at a density of 2 × 10^4^/well in 96-well plates and incubated at 37°C and 5% CO_2_ for 2 days until confluency.

### Candidalysin neutralization assays

Two clones of anti-candidalysin single-domain antibodies, specifically called V_H_H, but often referred to as nanobody (which is a registered trademark of Ablynx), CAL1-H1 and CAL1-F1 were generated against synthetic and native candidalysin, respectively, as described in reference (29). CAL1-H1 was produced in *Escherichia coli* BL21 and purified from the periplasmic extracts by immobilized metal affinity chromatography on the hist-tag, while CAL1-F1 was produced in *Saccharomyces cerevisiae* and purified by affinity chromatography using protA columns. Experiments were first performed on OECs using the *C. albicans* SC5314-derived BWP17/CIP30 wild-type strain at a final concentration of 2 × 10^4^ cells/well (MOI 1) or 16 µM candidalysin (Peptide Synthetics) in a 96-well plate with a final volume of 200 µL. Strains or peptide toxin were pre-incubated with serial dilutions (4, 8, and 16 µM) of the nanobodies CAL1-F1 or CAL1-H1 for 1 h at 37°C. All mixtures were prepared in serum-free cell culture media. After pre-incubation, mixtures were added to TR146 cells, previously washed 1× with the respective serum-free media. As a control nanobody that does not bind candidalysin, we included a V_H_H nanobody, anti-human epidermal growth factor receptor 2.

For neutralization assays on VECs, some modifications were applied. We used *C. albicans* SC5314 and candidalysin at a highly lytic concentration of 70 µM (48) to test the maximum neutralization potential of the nanobody. Nanobodies were either pre-incubated for 1 h at 37°C and shaking at 180 rpm with candidalysin or *C. albicans*, simultaneously added with candidalysin or *C. albicans*, or added 3 h after candidalysin treatment or *C. albicans* infection.

To determine the effect of anti-candidalysin nanobodies on cytokines released by OECs, epithelial cells were seeded at a density of 2.5 × 10^5^/well in 24-well plates, left to reach confluency until the next day, and serum-starved ON before infection. *C. albicans* SC5314 (MOI 0.01) was pre-incubated with 4 µM CAL1-F1 for 1 h at 37°C while shaking before the mixture was added to OECs in 24-well plates.

After 24 h incubation at 37°C and 5% CO_2_, plates were centrifuged for 10 min at 250 × *g*, and supernatants were collected for cytotoxicity and cytokine measurements.

### Host cell damage (cytotoxicity)

Epithelial cell damage was quantified by measuring the activity of the cytoplasmic enzyme LDH in the supernatant using a cytotoxicity detection kit (Roche) according to the manufacturer’s instructions.

### Quantification of calcium influx

OECs were seeded and serum starved before experiments. The following day, serum-free media were removed from the cells. A solution containing 2.5 µM Fura-2 AM (Thermo Scientific) and 500 µM probenecid (Sigma) was prepared in a saline solution (140 mM NaCl, 5 mM KCl, 1 mM MgCl_2_, 2 mM CaCl_2_, 10 mM glucose, and 10 mM HEPES [pH 7.4]). Cells were incubated with the solution containing Fura-2 AM for 1 h at 37°C and 5% CO_2_ in the dark. In the meantime, candidalysin (70 µM) and nanobody (4 and 16 µM, respectively) mix were prepared in saline solution in a 96-well plate. After sealing the plate, the plate was placed on a microplate shaker in a 37°C incubator for 1 h. Following the 1 h incubation steps, Fura-2 AM solution was removed from OECs, and the saline solution with or without candidalysin and nanobodies was added before readings were taken on a FlexStation 3 multimode microplate reader. Samples were excited at 340/380  nm, and fluorescence was detected at 520 nm. Readings were taken every minute. Results were expressed as a ratio between 340 and 380  nm. Data were normalized to OEC-only controls.

### Quantification of bilayer permeabilization

Current measurements were performed using the Orbit 16 system (Nanion) as described previously (48). In brief, the horizontal bilayers were formed using 1,2-diphytanoyl-sn-glycero-3-phosphocholine lipids in an electrolyte solution containing 0.1 M KCl and 20 mM HEPES at pH 7.4. Candidalysin peptides dissolved in water were added to bilayers at a final concentration of 10 µM. To monitor the effect of anti-candidalysin nanobodies, nanobody was pre-incubated with candidalysin before adding to the bilayer (molar ratio 1:1). Current changes were monitored at a constant voltage of −50 mV for 25 min using Element Data Recorder software (EDR v.3.8.3). Latencies until the membrane permeabilization were quantified using Clampfit v.10.3 (Molecular Devices).

### Lysate preparation and western blotting

For western blot experiments, OECs were seeded at a density of 2.5 × 10^5^/well in 24-well plates, left to reach confluency until the next day, and serum-starved ON before infection. *C. albicans* SC5314 was adjusted to MOI 10 and incubated together with 4 µM CAL1-F1 nanobody for 1 h at 37°C on a shaker before the mixture was added to OECs and incubated for 2 h at 37°C and 5% CO_2_. Following infections, tissue culture plates were placed on ice; culture medium was removed; and cells were washed with ice-cold PBS. Cells were lysed with 120 µL of RIPA buffer (25 mM Tris-HCl, pH 7.4, 150 mM NaCl, 1% Nonidet P-40, 1 mM EDTA, and 5% glycerol) supplemented with protease and phosphatase inhibitors (1:100 dilution, Sigma-Aldrich). Adherent cells were then scraped, transferred into pre-cooled microfuge tubes, and incubated on ice for 30 min. Lysates were clarified by centrifugation at 13,300 × *g* at 4°C for 10 min. Protein extract concentration was measured using a bicinchoninic acid assay (Thermo Fisher Scientific) according to the manufacturer’s instructions.

Proteins were resolved by electrophoresis on 20% SDS-PAGE gels. Following electrophoresis, proteins were transferred onto nitrocellulose membranes (Bio-Rad). Membranes were blocked in 1× Tris-buffered saline (TBST, Severn Biotech) containing 0.001% Tween 20 (Acros Organics) and 5% skimmed milk powder (Sainsbury’s). After washing once with TBST, membranes were incubated with primary antibody (Table S2) and gentle agitation ON at 4°C. The following day, membranes were washed three times for 5 min with TBST. Membranes were subsequently incubated with rabbit or mouse secondary antibody (Thermo Fisher Scientific) for 1 h at room temperature (RT) and then washed six times for 5 min with TBST. Finally, the proteins were detected using Immobilon Western Chemiluminescent HRP Substrate (Merck Millipore) and developed with an Odyssey Fc Imaging System (LI-COR). Human α-actin was used as a loading control.

### *ECE1* expression

To quantify *ECE1* mRNA expression, A-431 VECs were seeded in six-well plates at a density of 3 × 10^5^/well. After 2 days, confluent VECs were infected with 3 × 10^5^ *C. albicans* SC5314 cells (MOI 1) in the presence and absence of 4 µM CAL1-F1 nanobody for 24 h. The supernatant was removed from the wells, and 500 µL of RNeasy Lysis (RLT) buffer (QIAGEN) with 1% β-mercaptoethanol (Roth) was added. The well contents were detached using a cell scraper, frozen in liquid nitrogen, and stored at −80  °C until further use. *C. albicans* cells used as inoculum served as a 0 h control.

RNA was extracted by thawing the samples on ice and centrifugation for 10 min (20,000 × *g*, 4°C). Fungal RNA was isolated from the pellet using a freezing-thawing method, as described previously (60). RNA concentrations were measured with a NanoDrop 1000 Spectrophotometer (Thermo Fisher Scientific), and quality was assessed using an Agilent 2100 Bioanalyzer (Agilent Technologies). RNA (500 ng) was then treated with DNase I (Fermentas) following the manufacturer’s instructions and transcribed into complementary DNA (cDNA) using 0.5 µg of Oligo(dT)_12–18_ Primer, 200 U of Superscript III Reverse Transcriptase, and 40 U of RNaseOUT Recombinant RNase Inhibitor (Thermo Fischer Scientific). cDNA was diluted and used for qPCR with GoTaq qPCR Master Mix (Promega Corporation) in a CFX96 thermocycler (Bio-Rad Laboratories). Expression levels were normalized to the housekeeping gene *ACT1* (β-actin) and expressed relative to the expression of the target gene *ECE1* in 0 h control (log_2_ fold change). Primers that were used are listed in Table S3.

### Live-cell imaging of vaginal epithelial cell death

PI (Sigma-Aldrich) was used to stain non-viable VECs and monitor necrotic cell death over 24 h, as described previously (61). In brief, VECs were seeded in a 96-well plate, washed once with serum-free RPMI medium, and infected with 1  ×  10^5^ *C. albicans* (MOI 1) in medium with and without 4 µM CAL1-F1. PI was added to the wells at a final concentration of 4  µg/mL. VECs were imaged in a Zeiss Celldiscoverer 7 for 24 h at 37°C and 5% CO_2_. Images were taken every 20  min at ×10 magnification in bright field and fluorescence (excitation: 545  nm, emission: 572  nm). Using the threshold function in Fiji(62) images from the red fluorescence channel were converted to binary images. Macro batch analysis and the Particle Analyzer tool were used to quantify the number of PI-positive nuclei. The percentage of dead VECs was calculated in relation to the number of maximum dead host cells after 24 h.

### Imaging *C. albicans* hyphae

To determine if the anti-candidalysin nanobodies affect *C. albicans* hyphae, VECs were seeded in 24-well plates at a density of 1 × 10^5^/well. After 2 days, VECs were washed once with serum-free RPMI and infected with 1 × 10^5^ *C. albicans* (MOI 1) in the absence and presence of 4 µM CAL1-F1. After 6 h of incubation at 37°C and 5% CO_2_, VECs were washed once with PBS and fixed in 4% Histofix (Carl Roth). Bright field microscopy images were taken at ×20 magnification using a Zeiss Celldiscoverer v.7.

### Immunofluorescence staining for localization of nanobodies

For candidalysin immunofluorescence staining, A-431 VECs were seeded on glass coverslips (Ø 25 mm, VWR) in six-well plates at a density of 3 × 10^5^/well. To localize the anti-candidalysin nanobodies, VECs were treated with nanobodies at the onset of infection or 3 h after infection and stained. A-431 VECs were washed with RPMI-1640 medium and infected with 1.5 × 10^5^ *C. albicans* cells (MOI 0.5). After 3 h of incubation at 37°C and 5% CO_2_, the medium was removed, and the samples were fixed in 4% Histofix (Carl Roth). When the anti-candidalysin nanobody was added 3 h after infection, it was left to interact with secreted candidalysin for 15 min at 37°C and 5% CO_2_ prior to fixation. VECs were washed twice with PBS and incubated with concanavalin A-Alexa Fluor 647 (20 µg/mL in PBS, Invitrogen) in the dark for 30 min at RT. The cells were then washed twice with PBS, permeabilized with 0.01% Triton X-100 (Carl Roth) in PBS for 10 min at RT and washed once more with PBS. Samples were blocked with 0.5% bovine serum albumin (BSA) in PBS for 1 h at RT and washed twice with PBS. After washing with PBS, the secondary antibody goat IgG anti-camelid V_H_H (nanobody)-Alexa Fluor 488 (1:340 in 0.5% BSA in PBS, Jackson ImmunoResearch) was added and incubated for 1 h at RT. Samples were washed twice with PBS, mounted using SlowFade Diamond Antifade (Invitrogen), and visualized using fluorescence microscopy.

LSM 980 confocal microscope with Airyscan 2 detector (Carl Zeiss) equipped with C Plan-Apochromat ×63/NA 1.40 Oil DIC M27 objective lens was used to acquire high-resolved images of fungal and epithelial cells. The lasers at 488 and 639 nm were selected for fluorescence excitation of Alexa Fluor 488 and Alexa Fluor 647 dyes, respectively. An automated alignment was performed to calibrate the Airyscan detector before proceeding with the acquisition phase. The super resolution mode of the Airyscan was used with a calibration of 0.043 µm/pixel and a z-step of 0.170 µm. Fluorescence was detected with an Airyscan detector and super-resolution mode. The reconstruction was done using the Airyscan data processing included in the ZEN software with the automatic strength. All measurements were performed at RT. The images were saved in .lsm file format and then analyzed using Fiji/ImageJ.

### Isolation and stimulation of neutrophils

Primary human neutrophils were isolated as previously described (63) In brief, peripheral blood mononuclear cells were separated from granulocytes and erythrocytes using density gradient centrifugation over Histopaque-1077 (Sigma-Aldrich) in a 50-mL sterile tube. Neutrophils were isolated from the erythrocyte/granulocyte fraction using hypotonic lysis of erythrocytes in 155 mM NH_4_Cl and 10 mM KHCO_3_. Afterward, neutrophils were washed twice in PBS, resuspended in RPMI-1640 media, and seeded in a 96-well plate at a density of 5 × 10^4^–1 × 10^5^/well. Neutrophils were stimulated with supernatants (2× diluted in fresh RPMI) of VECs exposed to *C. albicans* with and without nanobody for 24 h as described above. As control, neutrophils were stimulated with CAL1-F1 nanobody (4 µM) alone. After stimulation, neutrophil supernatants were collected, and IL-8, an indicator of neutrophil activation, was measured using human enzyme-linked immunosorbent assays (R&D Systems) according to the manufacturer’s instructions.

Neutrophil activation was also assessed by flow cytometry. Neutrophils were seeded in a round bottom 96 well-plate at a density of 2 × 10^5^/well and stimulated with undiluted VEC supernatants and CAL1-F1 nanobody (4 µM) alone as control. Supernatants were removed after 3 h, and cells were washed in flow cytometry buffer (PBS, 2% fetal calf serum), which all consequent steps were performed in. To prevent unspecific staining, neutrophils were pre-incubated with Fc-Block Human TruStain FcX (BioLegend) before adding a mix of fluorophore-linked antibodies against the following activation status indicating surface molecules: CD11b-BV421 (ICR44), CD15-APC-Fire750 (W6D3), CD16-PerCP-Cy5.5 (3G8), CD35-FITC (E11), CD62L-AlexaFluor647 (DREG-56), CD66b-PE (G10F5), and CD182-PE-Cy7 (5E8, all from BioLegend). Activation markers were selected based on previous observations of granulocyte responses to fungal pathogens or associated stimuli (52, 54, 57). Fixable Viability Dye eFluor506 (Invitrogen) was used to exclude dead cells. Staining was performed for 20 min at 8°C. Afterward, cells were washed in flow cytometry buffer, filtered through a 70 µm mesh, and acquired on a FACSVerse Cell Analyzer flow cytometer (BD Biosciences). Analysis was performed in FlowJo v.10. For the gating strategy, see Fig. S6.

### Neutrophil staining and migration

Isolated primary human neutrophils were stained with cytopainter green (Abcam). Briefly, 2 µL of cytopainter green stock solution was added to 1 × 10^6^ neutrophils in RPMI and incubated at RT for 10 min in the dark. Stained neutrophils were washed once using Hank’s Balanced Salt Solution with 20 mM HEPES buffer (final pH 7) while being spun down at 300 × *g* for 10 min. After resuspension in endothelial cell medium (ECM, Promocell), the neutrophil cell number was determined using a Neubauer counting chamber.

Cryopreserved human umbilical cord vein endothelial cells (HUVECs) that were kindly provided by the lab of Alexander Mosig (University Clinic Jena) were expanded until four passages in 150-cm^2^ flasks using ECM and frozen in liquid nitrogen at a concentration of 1 × 10^6^/mL in ECM with 9% FBS and 7.5% dimethyl sulfoxide. HUVECs from glycerol stocks were cultured in 150-cm^2^ flasks for 72 h and harvested. Cells were then seeded at a density of 2 × 10^4^ cells in a transwell insert with a 3 µm pore size and incubated at 37°C with 5% CO_2_. After 48 h, transwell inserts were added to 24-well plates with confluent VECs that were seeded 2 days prior at a density of 1 × 10^5^ cells/well. Medium was refreshed in the transwell inserts (200 µL), and VECs were infected with 1 × 10^5^ *C. albicans* SC5314 cells (MOI 1) in the absence and presence of 4 µM anti-candidalysin nanobody (total volume 600 µL). Following 18 h of infection, 200 µL of cytopainter green-stained neutrophils (5 × 10^5^ cells/mL) was added to the transwell inserts. Plates were incubated for 2 h at 37°C and 5% CO_2_, where after images were taken of the wells using a Zeiss Celldiscoverer 7 (excitation: 493  nm, emission: 517  nm). The number of cytopainter green-positive events was determined using thresholding similar to that described above for PI image analysis. For each condition, neutrophil migration was quantified as a percentage of the total amount of neutrophils.

### Cytokine release

IL-1α, IL-6, IL-8, GM-CSF, and G-CSF were quantified in cell culture supernatants from OECs and candidalysin-treated VECs using magnetic microparticles (R&D Systems) with a magnetic Luminex performance assay (Bio-Techne) and a Bio-Plex 200 system (Bio-Rad) according to the manufacturers’ instructions. Data were analyzed using Bioplex Manager v.6.1 software. Supernatants from *C. albicans*-infected VECs were analyzed for additional cytokines (IL-8, IFN-α, IFN-β, CCL2, CCL3, CCL4, CCL5, CCL20, IL-1α, GM-CSF, G-CSF, IL-17, CXCL1, and CXCL2) using a multiplex human cytokine panel (R&D Systems) and the Luminex MAGPIX (Thermo Fisher Scientific) instrument according to the manufacturers’ instructions. Any other cytokines released were measured with commercially available human enzyme-linked immunosorbent assay kits (R&D Systems) according to the manufacturers’ instructions.

### Nanobody efficacy compared to fluconazole

The efficacy of the anti-candidalysin nanobody was compared to that of FLU, an azole frequently used to treat VVC. A-431 VECs were infected with 2 × 10^4^ *C. albicans* cells (MOI 1), and after 9 h, CAL1-F1 nanobody (4 µM) and/or FLU (4 µg/mL) was added. After a total of 24 h of infection at 37°C and 5% CO_2_, 96-well plates were spun down for 10 min at 250 × *g*, and supernatants were collected for cytotoxicity and cytokine measurements. To determine the combined effect of FLU (A) and nanobody (B) on epithelial cell damage, the CDI was calculated as AB/(A × B) using LDH data (64). After subtracting low controls AB, A, and B were expressed as fold change of the control group.

### *In silico* model description

Our *in silico* model is based on ordinary differential equations consisting of eight entities. All parameters and entities in the model are listed with their respective units in Table S4. Due to the fact that candidalysin needs to form multimeric aggregates to induce host cell lysis, the entity $C_{M}$ does not represent the concentration of a single monomer but a group of monomers consisting of the number of entities that are needed to form an aggregate. By making this assumption, the model effectively linearizes the process of aggregation or polymerization to simplify complex multimerization events. This linear approximation allows the model to capture the dynamics observed in the data while maintaining a manageable level of complexity.

The rate constant $k_{b}$ in the model’s first two equations is scaled by a factor of A, which ensures that the effective rate of association between the nanobody and A individual monomers of candidalysin supports the simplification of multimerization events:


(1) $$\frac{d\left[C_{M}\right]}{dt}\;=\;-k_{a}\;\left[C_{M}\right]\;–\frac{k_{b}}{A}\;\left[Nb\right]\;\left[C_{M}\right]\;+\;S\left(t\right),$$

(2) $$\frac{d\left[Nb\right]}{dt}\;=\;-k_{n}\left[Nb\right]\;–\;k_{b}\left[Nb\right]\left[C_{M}\right]–\;k_{b}\left[Nb\right]\left[C_{A}\right].$$

In these equations, parameters $k_{a}$ and $k_{n}$ represent the formation rate of the candidalysin aggregate and degradation rates of the nanobody, respectively. The function S(t) serves as a source term for candidalysin secretion by invasive filamentous *C. albicans* cells $\left(F_{I}\right)$ over time. While this function is not needed when modeling experiments with synthetic candidalysin, it is used to capture experimental data for *C. albicans*-secreted candidalysin. Parameter $k_{b}$ represents the blocking of candidalysin monomers and aggregates by anti-candidalysin nanobodies. The rate $k_{b}$ in model equations 1 and 2 is scaled by a factor of A to ensure that the effective rate of association between the nanobody and A individual monomers of candidalysin supports the model’s simplification with regard to aggregation representation. In addition, we assumed that the nanobody targets the candidalysin monomer and aggregate with the same binding affinity in a 1:1 ratio; i.e., the nanobody has the same binding affinity to the aggregate as to individual monomers.

The transformation of candidalysin into the aggregate through a reaction involving monomeric candidalysin and its depletion through a reaction involving VECs is given by


(3) $$\frac{d\left[C_{A}\right]}{dt}\;=\;k_{a}\left[C_{A}\right]\;-\;\alpha k_{d}\left[C_{A}\right]\left[E\right]–\;k_{b}\left[Nb\right]\left[C_{A}\right].$$

Here, $k_{d}$ is depletion of the candidalysin aggregate due to integration into the host cell membrane subsequently causing damage. The variable *α* is a conversion constant describing the amount of aggregated candidalysin being depleted relative to 1% of dead VECs.

The damage of VECs caused by aggregated candidalysin is given by


(4) $$\frac{d\left[E\right]}{dt}\;=\;-k_{d}\;\left[C_{A}\right]\left[E\right]$$

while the production of LDH due to damaged VECs and its degradation rate $k_{l}$ is given by


(5) $$\frac{d\left[LDH\right]}{dt}\;=\;\beta k_{d}\;\left[C_{A}\right]\left[E\right]-\;k_{l}\;\left[LDH\right].$$

Here, the parameter β is a conversion constant that describes the amount of LDH released upon host cell death.

To model the system with *C. albicans*-secreted candidalysin, we added three more equations to include invasion by *C. albicans* cells over time:


(6) $$\frac{d\left[Y\right]}{dt}\;=\;-r\;\left[Y\right],$$

(7) $$\frac{d\left[F_{NI}\right]}{dt}=\;r\;\left[Y\right]–\;r_{i}\;\left[F_{NI}\;\right],$$

(8) $$\frac{d\left[F_{I}\right]}{dt}=\;r_{i}\;\left[F_{NI}\right].$$

In these equations, r is the transition rate of yeast cells Y into non-invasive filamentous cells $F_{NI}$ and $r_{i}$ is the transition rate from $F_{NI}$ into invasive filamentous cells $F_{I}$ . These processes were modeled previously on OECs, and the corresponding parameters were estimated as previously described in detail (31).

Furthermore, the source term in eqation 1 was set to


(9) $$S\left(t\right)\;=\;k_{s}\;\left[F_{I}\right]\left[E\right],$$

where $k_{s}$ refers to the secretion rate of candidalysin. We modeled this process as an interaction term between alive VECs and filamentous invasive *C. albicans* cells. Therefore, only the candidalysin that was able to cause damage was secreted, which implies that $k_{s}$ is a secretion rate of “effective” candidalysin. Since the parameters $k_{a}$, $k_{d}$, and $k_{b}$ are taken from the synthetic candidalysin experiments, the parameter $k_{s}$ should be more understood as secretion of candidalysin that is equivalent to synthetic candidalysin.

### *In silico* model parameter estimation

Our *in silico* model incorporates various parameters (Table S4) that govern the temporal dynamics of the host damage marker LDH. These parameters cannot be directly observed; therefore, their estimation was based on fitting the model to *in vitro* experimental data (Fig. 2A through C; Fig. S2). To capture the dynamics observed in the data, we utilized a bottom-up approach. The model’s prediction discrepancy was evaluated by comparing it with (i) the LDH concentration and (ii) the percentage of dead VECs minimizing the sum of squared errors (SSE). The percentage of dead VECs is not directly present in the data, but as reference, we can define that this value should be at least as high as the LDH in the *in vitro* data as a fraction of maximum LDH release if all VECs are dead (estimated by β). This resulted into the following discrepancy measure:


(10) $$SSE\;=\;\sum_{i=1}^{n}\left[{\left({LDH}_{sim}^{i}\;-LDH_{data}^{i}\right)}^{2}+max{\left(\left(1-E_{sim}^{i}\right)-\frac{LDH_{data}^{i}}{\beta },0.0\right)}^{2}\right].$$

**Fig 6:**
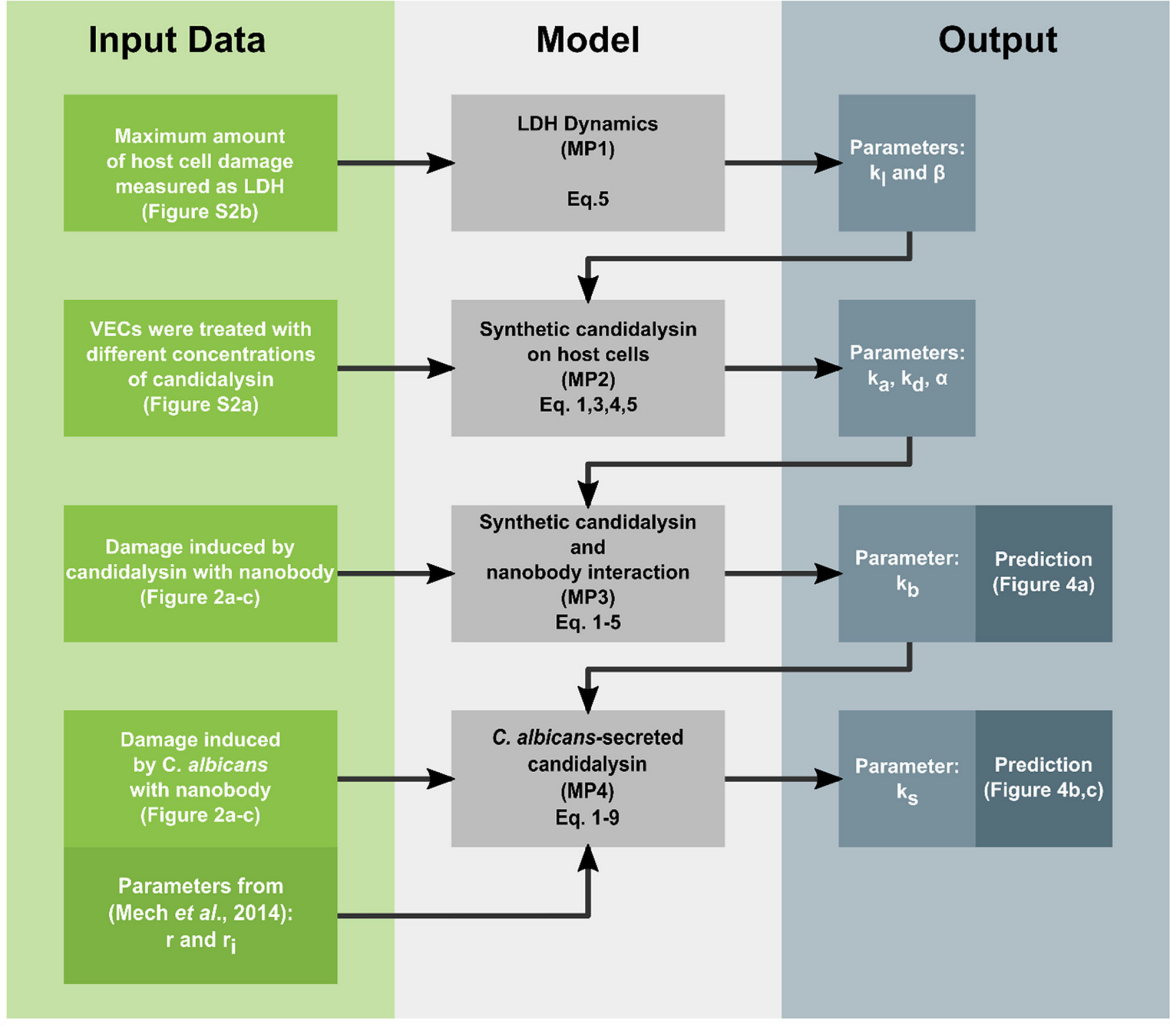
Schematic overview of our bottom-up approach for estimating the model parameters, given the *in vitro* experimental data.

Our modeling approach includes four model parts, each with different levels of complexity and interconnected hierarchically. The simplest model part (MP1) encapsulates only the dynamics related to LDH seen in equation 5, without the presence of candidalysin. The next model part (MP2) integrates candidalysin as a damage-causing toxin as given in equations 1 and 3-5, without the presence of the nanobody interaction. The more complex model part (MP3) incorporates blocking of candidalysin by the nanobody in equation 2, and the final model part (MP4) estimates *C. albicans*-related parameters by including equations 6-8. A full overview of the parameter estimation procedure is depicted in Fig. 6.

We fitted these models hierarchically using a bottom-up method, i.e., the estimates from the less complex model were used as fixed parameters for the more complex ones. Thus, we assumed that the nanobody and candidalysin interactions are comparable between synthetic and *C. albicans*-secreted candidalysin. Each model was fitted on a different data set vital for estimating the mechanisms of the associated data.

We employed the SciPy minimization package (65) with 10 million different initial values for each fit to accurately pinpoint the true global minimum. The initial values were sampled using Latin Hypercube.

A profile likelihood method (66) was used to assess the confidence interval for each parameter at a significance level of 0.05 surrounding the maximum-likelihood estimate. To evaluate the profile likelihood, we compared the chi-squared (χ^2^) test statistics at the 95% percentile (approximately 3.84) with the negative logarithm of the likelihood ratio multiplied by 2. This approach enabled us to assess the practical identifiability of all parameters in our model. Figure S7 illustrates the profiles of the likelihood function for each parameter that was used to obtain the confidence intervals.

### *In silico* model sensitivity analysis

A global Sobol sensitivity analysis was conducted using the open-source Python library SAlib (67, 68) to assess the impact and the nature of the influence of relevant parameters. The parameters were tested for their first and total order Sobol sensitivity in relation to the amount LDH at various time points. The analysis involved sampling 4,096 points uniformly from the confidence intervals obtained through the profile likelihood method depicted in Table 1 for each parameter.

### *In silico* model numerical simulation

The numerical simulation of our model was executed using the LSODA solver incorporated in the SciPy library. LSODA is a versatile and robust solver adept at efficiently handling stiff systems of ordinary differential equations. The solver numerically integrated the system of equations over time, starting from the initial conditions of $Nb,C_{A},Y$, and E. The integration was carried out over a time range of interest, specifically [0, 72], with the two parameters *atol* for the absolute error and *rtol* for the relative error set to 1e-9 and 1e-10, respectively. The resulting time-dependent profiles of the key molecular entities in the system were then analyzed and compared to experimental measurements (refer to parameter estimation).

## Data Availability

All the codes associated with the model description, including fitting procedure and prediction generation, are available in the GitHub repository. Additionally, the experimental data for the model fitting and intermediate results derived from the bottom-up fitting can be accessed at https://asbdata.hki-jena.de/ValentineEtAl2024_mBio.

## SUPPLEMENTAL MATERIAL

Supplemental material mbio.03409-23-s0001.pdf Fig. S1 to S7; Tables S1 to S4.

ASM does not own the copyrights to Supplemental Material that may be linked to, or accessed through, an article. The authors have granted ASM a non-exclusive, world-wide license to publish the Supplemental Material files. Please contact the corresponding author directly for reuse.

## References

[1] Drell T, Lillsaar T, Tummeleht L, Simm J, Aaspõllu A, Väin E, Saarma I, Salumets A, Donders GGG, Metsis M. 2013. Characterization of the vaginal micro- and mycobiome in asymptomatic reproductive-age Estonian women. PLoS One 8:e54379. doi:10.1371/journal.pone.0054379 23372716 PMC3553157

[2] Nash AK, Auchtung TA, Wong MC, Smith DP, Gesell JR, Ross MC, Stewart CJ, Metcalf GA, Muzny DM, Gibbs RA, Ajami NJ, Petrosino JF. 2017. The gut mycobiome of the human microbiome project healthy cohort. Microbiome 5:153. doi:10.1186/s40168-017-0373-4 29178920 PMC5702186

[3] Ghannoum MA, Jurevic RJ, Mukherjee PK, Cui F, Sikaroodi M, Naqvi A, Gillevet PM. 2010. Characterization of the oral fungal microbiome (mycobiome) in healthy individuals. PLoS Pathog 6:e1000713. doi:10.1371/journal.ppat.1000713 20072605 PMC2795202

[4] d’Enfert C, Kaune A-K, Alaban L-R, Chakraborty S, Cole N, Delavy M, Kosmala D, Marsaux B, Fróis-Martins R, Morelli M, et al.. 2021. The impact of the fungus-host-microbiota interplay upon Candida albicans infections: current knowledge and new perspectives. FEMS Microbiol Rev 45:fuaa060. doi:10.1093/femsre/fuaa060 33232448 PMC8100220

[5] Samaranayake LP. 1992. Oral mycoses in HIV infection. Oral Surg Oral Med Oral Pathol 73:171–180. doi:10.1016/0030-4220(92)90191-r 1549312

[6] Sobel JD. 2007. Vulvovaginal candidosis. Lancet 369:1961–1971. doi:10.1016/S0140-6736(07)60917-9 17560449

[7] Yano J, Sobel JD, Nyirjesy P, Sobel R, Williams VL, Yu Q, Noverr MC, Fidel PL. 2019. Current patient perspectives of vulvovaginal candidiasis: incidence, symptoms, management and post-treatment outcomes. BMC Womens Health 19:48. doi:10.1186/s12905-019-0748-8 30925872 PMC6441174

[8] Denning DW, Kneale M, Sobel JD, Rautemaa-Richardson R. 2018. Global burden of recurrent vulvovaginal candidiasis: a systematic review. Lancet Infect Dis 18:e339–e347. doi:10.1016/S1473-3099(18)30103-8 30078662

[9] Mayer FL, Wilson D, Hube B. 2013. Candida albicans pathogenicity mechanisms. Virulence 4:119–128. doi:10.4161/viru.22913 23302789 PMC3654610

[10] Richardson JP, Willems HME, Moyes DL, Shoaie S, Barker KS, Tan SL, Palmer GE, Hube B, Naglik JR, Peters BM. 2018. Candidalysin drives epithelial signaling, neutrophil recruitment, and immunopathology at the vaginal mucosa. Infect Immun 86:e00645-17. doi:10.1128/IAI.00645-17 29109176 PMC5778364

[11] Moyes DL, Wilson D, Richardson JP, Mogavero S, Tang SX, Wernecke J, Höfs S, Gratacap RL, Robbins J, Runglall M, et al.. 2016. Candidalysin is a fungal peptide toxin critical for mucosal infection. Nature 532:64–68. doi:10.1038/nature17625 27027296 PMC4851236

[12] Pekmezovic M, Hovhannisyan H, Gresnigt MS, Iracane E, Oliveira-Pacheco J, Siscar-Lewin S, Seemann E, Qualmann B, Kalkreuter T, Müller S, Kamradt T, Mogavero S, Brunke S, Butler G, Gabaldón T, Hube B. 2021. Candida pathogens induce protective mitochondria-associated type I interferon signalling and a damage-driven response in vaginal epithelial cells. Nat Microbiol 6:643–657. doi:10.1038/s41564-021-00875-2 33753919

[13] Naglik JR, Gaffen SL, Hube B. 2019. Candidalysin: discovery and function in Candida albicans infections. Curr Opin Microbiol 52:100–109. doi:10.1016/j.mib.2019.06.002 31288097 PMC6687503

[14] Blagojevic M, Camilli G, Maxson M, Hube B, Moyes DL, Richardson JP, Naglik JR. 2021. Candidalysin triggers epithelial cellular stresses that induce necrotic death. Cell Microbiol 23:e13371. doi:10.1111/cmi.13371 34085369 PMC8460601

[15] Westman J, Plumb J, Licht A, Yang M, Allert S, Naglik JR, Hube B, Grinstein S, Maxson ME. 2022. Calcium-dependent ESCRT recruitment and lysosome exocytosis maintain epithelial integrity during Candida albicans invasion. Cell Reports 38:110187. doi:10.1016/j.celrep.2021.110187 34986345 PMC8755444

[16] Wilson D, Naglik JR, Hube B, Hogan DA. 2016. The missing link between Candida albicans hyphal morphogenesis and host cell damage. PLoS Pathog 12:e1005867. doi:10.1371/journal.ppat.1005867 27764260 PMC5072684

[17] Mori T, Kataoka H, Tanabe G, Into T. 2022. Solubility affects IL-1beta-producing activity of the synthetic candidalysin peptide. PLoS One 17:e0273663. doi:10.1371/journal.pone.0273663 36040970 PMC9426886

[18] Ho J, Yang X, Nikou SA, Kichik N, Donkin A, Ponde NO, Richardson JP, Gratacap RL, Archambault LS, Zwirner CP, Murciano C, Henley-Smith R, Thavaraj S, Tynan CJ, Gaffen SL, Hube B, Wheeler RT, Moyes DL, Naglik JR. 2019. Candidalysin activates innate epithelial immune responses via epidermal growth factor receptor. Nat Commun 10:2297. doi:10.1038/s41467-019-09915-2 31127085 PMC6534540

[19] Moyes DL, Runglall M, Murciano C, Shen C, Nayar D, Thavaraj S, Kohli A, Islam A, Mora-Montes H, Challacombe SJ, Naglik JR. 2010. A biphasic innate immune MAPK response discriminates between the yeast and hyphal forms of Candida albicans in epithelial cells. Cell Host Microbe 8:225–235. doi:10.1016/j.chom.2010.08.002 20833374 PMC2991069

[20] Verma AH, Richardson JP, Zhou C, Coleman BM, Moyes DL, Ho J, Huppler AR, Ramani K, McGeachy MJ, Mufazalov IA, Waisman A, Kane LP, Biswas PS, Hube B, Naglik JR, Gaffen SL. 2017. Oral epithelial cells orchestrate innate type 17 responses to Candida albicans through the virulence factor candidalysin. Sci Immunol 2:eaam8834. doi:10.1126/sciimmunol.aam8834 29101209 PMC5881387

[21] Ho J, Wickramasinghe DN, Nikou SA, Hube B, Richardson JP, Naglik JR. 2020. Candidalysin is a potent trigger of alarmin and antimicrobial peptide release in epithelial cells. Cells 9:699. doi:10.3390/cells9030699 32178483 PMC7140650

[22] Ardizzoni A, Wheeler RT, Pericolini E. 2021. It takes two to tango: how a dysregulation of the innate immunity, coupled with Candida virulence, triggers VVC onset. Front Microbiol 12:692491. doi:10.3389/fmicb.2021.692491 34163460 PMC8215348

[23] Fidel PL, Barousse M, Espinosa T, Ficarra M, Sturtevant J, Martin DH, Quayle AJ, Dunlap K. 2004. An intravaginal live Candida challenge in humans leads to new hypotheses for the immunopathogenesis of vulvovaginal candidiasis. Infect Immun 72:2939–2946. doi:10.1128/IAI.72.5.2939-2946.2004 15102806 PMC387876

[24] Yano J, Peters BM, Noverr MC, Fidel PL, Maurelli AT. 2018. Novel mechanism behind the immunopathogenesis of vulvovaginal candidiasis: “neutrophil anergy”. Infect Immun 86:e00684-17. doi:10.1128/IAI.00684-17 PMC5820946 29203543

[25] Yano J, Noverr MC, Fidel PL, Pirofski L. 2017. Vaginal heparan sulfate linked to neutrophil dysfunction in the acute inflammatory response associated with experimental vulvovaginal candidiasis. mBio 8:e00211-17. doi:10.1128/mBio.00211-17 28292981 PMC5350465

[26] Ardizzoni A, Sala A, Colombari B, Giva LB, Cermelli C, Peppoloni S, Vecchiarelli A, Roselletti E, Blasi E, Wheeler RT, Pericolini E. 2020. Perinuclear anti-neutrophil cytoplasmic antibodies (pANCA) impair neutrophil candidacidal activity and are increased in the cellular fraction of vaginal samples from women with vulvovaginal candidiasis. J Fungi (Basel) 6:225. doi:10.3390/jof6040225 33081210 PMC7712103

[27] Naglik JR, König A, Hube B, Gaffen SL. 2017. Candida albicans-epithelial interactions and induction of mucosal innate immunity. Curr Opin Microbiol 40:104–112. doi:10.1016/j.mib.2017.10.030 29156234 PMC5733685

[28] Bruno VM, Shetty AC, Yano J, Fidel PL, Noverr MC, Peters BM. 2015. Transcriptomic analysis of vulvovaginal candidiasis identifies a role for the NLRP3 inflammasome. mBio 6:e00182-15. doi:10.1128/mBio.00182-15 25900651 PMC4453569

[29] Mogavero S, Sauer FM, Brunke S, Allert S, Schulz D, Wisgott S, Jablonowski N, Elshafee O, Krüger T, Kniemeyer O, Brakhage AA, Naglik JR, Dolk E, Hube B. 2021. Candidalysin delivery to the invasion pocket is critical for host epithelial damage induced by Candida albicans. Cell Microbiol 23:e13378. doi:10.1111/cmi.13378 34245079 PMC8460606

[30] Russell CM, Schaefer KG, Dixson A, Gray ALH, Pyron RJ, Alves DS, Moore N, Conley EA, Schuck RJ, White TA, Do TD, King GM, Barrera FN. 2022. The Candida albicans virulence factor candidalysin polymerizes in solution to form membrane pores and damage epithelial cells. Elife 11:e75490. doi:10.7554/eLife.75490 36173096 PMC9522247

[31] Mech F, Wilson D, Lehnert T, Hube B, Thilo Figge M. 2014. Epithelial invasion outcompetes hypha development during Candida albicans infection as revealed by an image-based systems biology approach. Cytometry A 85:126–139. doi:10.1002/cyto.a.22418 24259441

[32] Zhang XY, Trame MN, Lesko LJ, Schmidt S. 2015. Sobol sensitivity analysis: a tool to guide the development and evaluation of systems pharmacology models. CPT Pharmacometrics Syst Pharmacol 4:69–79. doi:10.1002/psp4.6 27548289 PMC5006244

[33] Pleckaityte M, Mistiniene E, Lasickiene R, Zvirblis G, Zvirbliene A. 2011. Generation of recombinant single-chain antibodies neutralizing the cytolytic activity of vaginolysin, the main virulence factor of Gardnerella vaginalis. BMC Biotechnol 11:100. doi:10.1186/1472-6750-11-100 22047084 PMC3226441

[34] Bernedo-Navarro RA, Romão E, Yano T, Pinto J, De Greve H, Sterckx YG-J, Muyldermans S. 2018. Structural basis for the specific neutralization of Stx2A with a camelid single domain antibody fragment. Toxins (Basel) 10:108. doi:10.3390/toxins10030108 29494518 PMC5869396

[35] McClelland EE, Bernhardt P, Casadevall A. 2005. Coping with multiple virulence factors: which is most important? PLoS Pathog 1:e40. doi:10.1371/journal.ppat.0010040 16738705 PMC1323474

[36] Casadevall A, Pirofski LA. 2009. Virulence factors and their mechanisms of action: the view from a damage-response framework. J Water Health 7 Suppl 1:S2–S18. doi:10.2166/wh.2009.036 19717929

[37] Vij R, Hube B, Brunke S. 2021. Uncharted territories in the discovery of antifungal and antivirulence natural products from bacteria. Comput Struct Biotechnol J 19:1244–1252. doi:10.1016/j.csbj.2021.02.003 33680363 PMC7905183

[38] Allen RC, Popat R, Diggle SP, Brown SP. 2014. Targeting virulence: can we make evolution-proof drugs Nat Rev Microbiol 12:300–308. doi:10.1038/nrmicro3232 24625893

[39] Roselletti E, Pericolini E, Nore A, Takacs P, Kozma B, Sala A, De Seta F, Comar M, Usher J, Brown GD, Wilson D. 2023. Zinc prevents vaginal candidiasis by inhibiting expression of an inflammatory fungal protein. Sci Transl Med 15:eadi3363. doi:10.1126/scitranslmed.adi3363 38055800 PMC7616067

[40] Farr A, Effendy I, Frey Tirri B, Hof H, Mayser P, Petricevic L, Ruhnke M, Schaller M, Schaefer APA, Sustr V, Willinger B, Mendling W. 2021. Guideline: vulvovaginal candidosis (AWMF 015/072, level S2K). Mycoses 64:583–602. doi:10.1111/myc.13248 33529414 PMC8248160

[41] Donders G, Bellen G, Byttebier G, Verguts L, Hinoul P, Walckiers R, Stalpaert M, Vereecken A, Van Eldere J. 2008. Individualized decreasing-dose maintenance fluconazole regimen for recurrent vulvovaginal candidiasis (ReCiDiF trial). Am J Obstet Gynecol 199:613. doi:10.1016/j.ajog.2008.06.029 18976735

[42] Neal CM, Martens MG. 2022. Clinical challenges in diagnosis and treatment of recurrent vulvovaginal candidiasis. SAGE Open Med 10:20503121221115201. doi:10.1177/20503121221115201 36105548 PMC9465564

[43] Gaziano R, Sabbatini S, Roselletti E, Perito S, Monari C. 2020. Saccharomyces cerevisiae-based probiotics as novel antimicrobial agents to prevent and treat vaginal infections. Front Microbiol 11:718. doi:10.3389/fmicb.2020.00718 32373104 PMC7186379

[44] Makanjuola O, Bongomin F, Fayemiwo SA. 2018. An update on the roles of non-albicans Candida species in vulvovaginitis. JoF 4:121. doi:10.3390/jof4040121 30384449 PMC6309050

[45] König A, Hube B, Kasper L. 2020. The dual function of the fungal toxin candidalysin during Candida albicans-macrophage interaction and virulence. Toxins 12:469. doi:10.3390/toxins12080469 32722029 PMC7471981

[46] Willems HME, Lowes DJ, Barker KS, Palmer GE, Peters BM. 2018. Comparative analysis of the capacity of the Candida species to elicit vaginal immunopathology. Infect Immun 86:e00527-18. doi:10.1128/IAI.00527-18 30249743 PMC6246903

[47] Russell CM, Rybak JA, Miao J, Peters BM, Barrera FN. 2023. Candidalysin: connecting the pore forming mechanism of this virulence factor to its immunostimulatory properties. J Biol Chem 299:102829. doi:10.1016/j.jbc.2022.102829 36581211 PMC9852700

[48] Richardson JP, Brown R, Kichik N, Lee S, Priest E, Mogavero S, Maufrais C, Wickramasinghe DN, Tsavou A, Kotowicz NK, Hepworth OW, Gallego-Cortés A, Ponde NO, Ho J, Moyes DL, Wilson D, D’Enfert C, Hube B, Naglik JR. 2022. Candidalysins are a new family of cytolytic fungal peptide toxins. mBio 13:e0351021. doi:10.1128/mbio.03510-21 35073742 PMC8787473

[49] Liu J, Willems HME, Sansevere EA, Allert S, Barker KS, Lowes DJ, Dixson AC, Xu Z, Miao J, DeJarnette C, Tournu H, Palmer GE, Richardson JP, Barrera FN, Hube B, Naglik JR, Peters BM, Wheeler RT. 2021. A variant ECE1 allele contributes to reduced pathogenicity of Candida albicans during vulvovaginal candidiasis. PLoS Pathog 17:e1009884. doi:10.1371/journal.ppat.1009884 34506615 PMC8432879

[50] Zhu W, Phan QT, Boontheung P, Solis NV, Loo JA, Filler SG. 2012. EGFR and HER2 receptor kinase signaling mediate epithelial cell invasion by Candida albicans during oropharyngeal infection. Proc Natl Acad Sci U S A 109:14194–14199. doi:10.1073/pnas.1117676109 22891338 PMC3435201

[51] Hanaoka M, Domae E. 2021. IL-1α released from oral epithelial cells upon candidalysin exposure initiates an early innate epithelial response. Int Immunol 33:161–170. doi:10.1093/intimm/dxaa070 33038250

[52] Sabroe I, Jones EC, Whyte MKB, Dower SK. 2005. Regulation of human neutrophil chemokine receptor expression and function by activation of toll-like receptors 2 and 4. Immunology 115:90–98. doi:10.1111/j.1365-2567.2005.02133.x 15819701 PMC1782127

[53] Simon SI, Green CE. 2005. Molecular mechanics and dynamics of leukocyte recruitment during inflammation. Annu Rev Biomed Eng 7:151–185. doi:10.1146/annurev.bioeng.7.060804.100423 16004569

[54] Dietschmann A, Schruefer S, Westermann S, Henkel F, Castiglione K, Willebrand R, Adam J, Ruland J, Lang R, Sheppard DC, Esser-von-Bieren J, Radtke D, Krappmann S, Voehringer D. 2022. Phosphatidylinositol 3-kinase (Pi3K) orchestrates Aspergillus fumigatus-induced eosinophil activation independently of canonical toll-like receptor (TLR)/C-type-lectin receptor (CLR) signaling. mBio 13:e0123922. doi:10.1128/mbio.01239-22 35695427 PMC9426586

[55] Kishimoto TK, Jutila MA, Berg EL, Butcher EC. 1989. Neutrophil Mac-1 and MEL-14 adhesion proteins inversely regulated by chemotactic factors. Science 245:1238–1241. doi:10.1126/science.2551036 2551036

[56] Griffin JD, Spertini O, Ernst TJ, Belvin MP, Levine HB, Kanakura Y, Tedder TF. 1990. Granulocyte-macrophage colony-stimulating factor and other cytokines regulate surface expression of the leukocyte adhesion molecule-1 on human neutrophils, monocytes, and their precursors. J Immunol 145:576–584. 1694883

[57] Simard JC, Girard D, Tessier PA. 2010. Induction of neutrophil degranulation by S100A9 via a MAPK-dependent mechanism. J Leukoc Biol 87:905–914. doi:10.1189/jlb.1009676 20103766

[58] Avril A, Tournier JN, Paucod JC, Fournes B, Thullier P, Pelat T. 2022. Antibodies against anthrax toxins: a long way from benchlab to the bedside. Toxins (Basel) 14:172. doi:10.3390/toxins14030172 35324669 PMC8955606

[59] Gillum AM, Tsay EY, Kirsch DR. 1984. Isolation of the Candida albicans gene for orotidine-5'-phosphate decarboxylase by complementation of S. cerevisiae ura3 and E. coli pyrF mutations. Mol Gen Genet 198:179–182. doi:10.1007/BF00328721 6394964

[60] Wächtler B, Wilson D, Haedicke K, Dalle F, Hube B. 2011. From attachment to damage: defined genes of Candida albicans mediate adhesion, invasion and damage during interaction with oral epithelial cells. PLoS One 6:e17046. doi:10.1371/journal.pone.0017046 21407800 PMC3044159

[61] Austermeier S, Pekmezović M, Porschitz P, Lee S, Kichik N, Moyes DL, Ho J, Kotowicz NK, Naglik JR, Hube B, Gresnigt MS, Cowen LE. 2021. Albumin neutralizes hydrophobic toxins and modulates Candida albicans pathogenicity. mBio 12:e0053121. doi:10.1128/mBio.00531-21 34154403 PMC8262992

[62] Schindelin J, Arganda-Carreras I, Frise E, Kaynig V, Longair M, Pietzsch T, Preibisch S, Rueden C, Saalfeld S, Schmid B, Tinevez J-Y, White DJ, Hartenstein V, Eliceiri K, Tomancak P, Cardona A. 2012. Fiji: an open-source platform for biological-image analysis. Nature methods 9:676–682. doi:10.1038/nmeth.2019 22743772 PMC3855844

[63] Gresnigt MS, Joosten LAB, Verschueren I, van der Meer JWM, Netea MG, Dinarello CA, van de Veerdonk FL. 2012. Neutrophil-mediated inhibition of proinflammatory cytokine responses. J Immunol 189:4806–4815. doi:10.4049/jimmunol.1103551 23053514

[64] Chen L, Ye H-L, Zhang G, Yao W-M, Chen X-Z, Zhang F-C, Liang G, Ho Y-S. 2014. Autophagy inhibition contributes to the synergistic interaction between EGCG and doxorubicin to kill the hepatoma Hep3B cells. PLoS One 9:e85771. doi:10.1371/journal.pone.0085771 24465696 PMC3897495

[65] Virtanen P, Gommers R, Oliphant TE, Haberland M, Reddy T, Cournapeau D, Burovski E, Peterson P, Weckesser W, Bright J, et al.. 2020. SciPy 1.0: fundamental algorithms for scientific computing in python. Nat Methods 17:261–272. doi:10.1038/s41592-019-0686-2 32015543 PMC7056644

[66] Kreutz C, Raue A, Kaschek D, Timmer J. 2013. Profile likelihood in systems biology. FEBS J 280:2564–2571. doi:10.1111/febs.12276 23581573

[67] Iwanaga T, Usher W, Herman J. 2022. Toward SALiB 2.0: advancing the accessibility and interpretability of global sensitivity analyses. SESMO 4:18155. doi:10.18174/sesmo.18155

[68] Herman J, Usher W. 2017. SALiB: an open-source python library for sensitivity analysis. JOSS 2:97. doi:10.21105/joss.00097

