# Supplementary material for: Nanobody-mediated neutralization of candidalysin prevents epithelial damage and inflammatory responses that drive vulvovaginal candidiasis pathogenesis
Source: mBio. 2024 Feb 13;15(3):e03409-23. doi: 10.1128/mbio.03409-23 (PMC10936171; doi:10.1128/mbio.03409-23)
Supplement: Supplemental material — Fig. S1 to S7; Tables S1 to S4. [file mbio.03409-23-s0001.pdf]

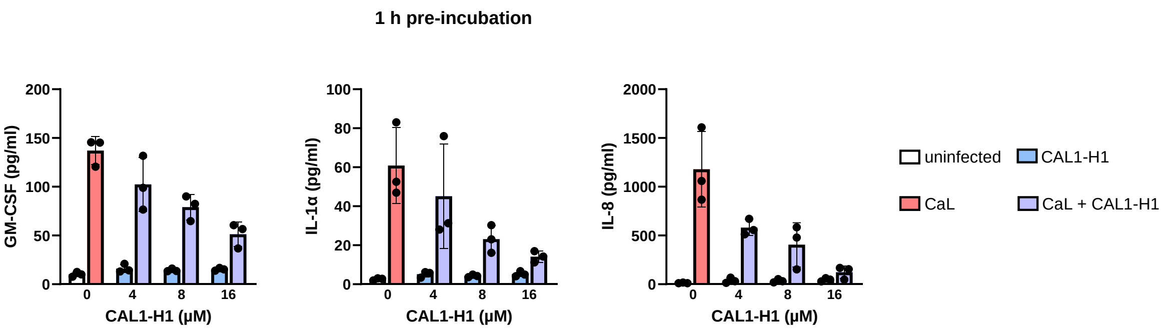

Figure S1. Anti-candidalysin nanobodies reduced cytokine secretion by candidalysin-treated A-431 vaginal epithelial cells (VECs). GM-CSF, IL-1α, and IL-8 secretion by VECs treated with 70 µM candidalysin (CaL) in the presence of increasing concentrations of CAL1-H1 anti-candidalysin nanobody (4, 8, and 16 µM) were measured. Nanobodies were added to VECs after pre-incubation with candidalysin for 1 h. Bars represent the mean ± standard deviation (SD) of *n* = 3 independent replicates. Means were compared to the candidalysin controls for significance using a One-way ANOVA with a Šidák's multiple comparisons test.

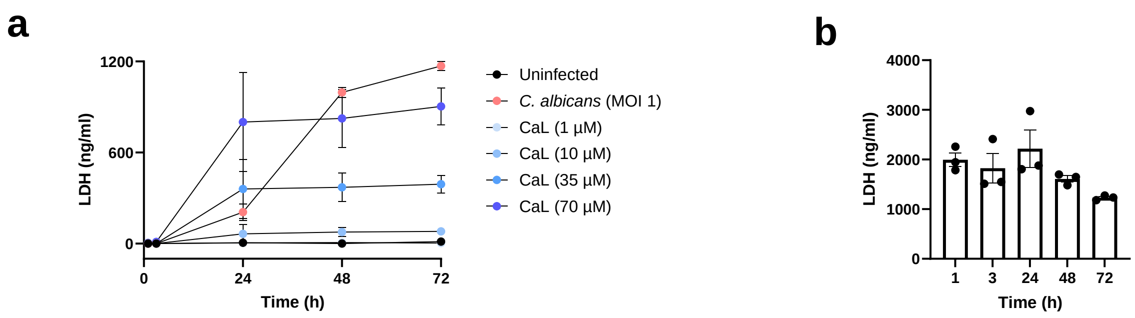

Figure S2. ***In vitro*** **data of vaginal epithelial cell (VEC) damage over time. (a)** VECs were treated with different concentrations of candidalysin (CaL) or infected with *Candida albicans* (MOI 1) and released lactate dehydrogenase (LDH) was measured after 1, 3, 24, 48, and 72 h to quantify host cell damage. **(b)** Confluent VECs were lysed using Triton X-100 to determine the maximum amount of host cell damage after 1, 3, 24, 48, and 72 h.

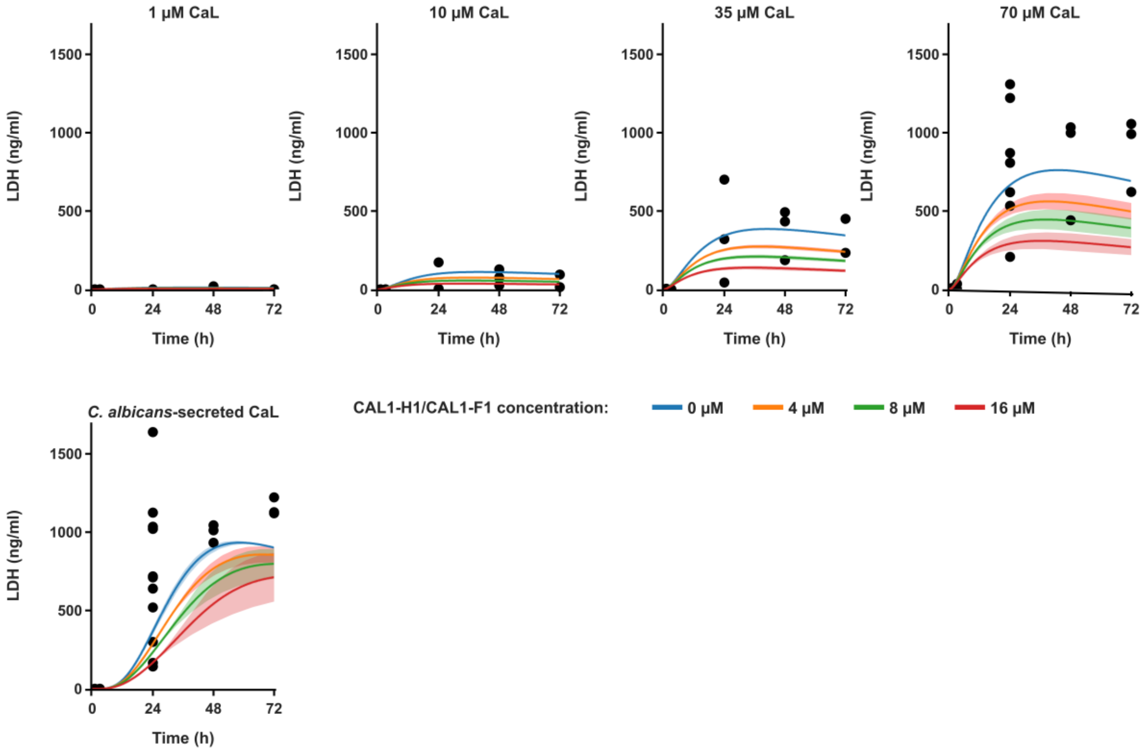

Figure S3. Model fit of *in vitro* experimental data with candidalysin and anti-candidalysin nanobodies. *In silico* model results for 72 h for varying candidalysin (CaL) concentrations and *C. albicans*-secreted candidalysin. Black dots indicate *in vitro* experimental data points without nanobody. Similar to *in vitro* data, CAL1-H1 was used to model neutralization of synthetic candidalysin, while CAL1-F1 was used to model neutralization of *C. albicans*-secreted candidalysin. Curves indicate *in silico* estimated dynamic of lactate dehydrogenase (LDH) release. Error bands across all plots depict the variability from varying candidalysin aggregate sizes.

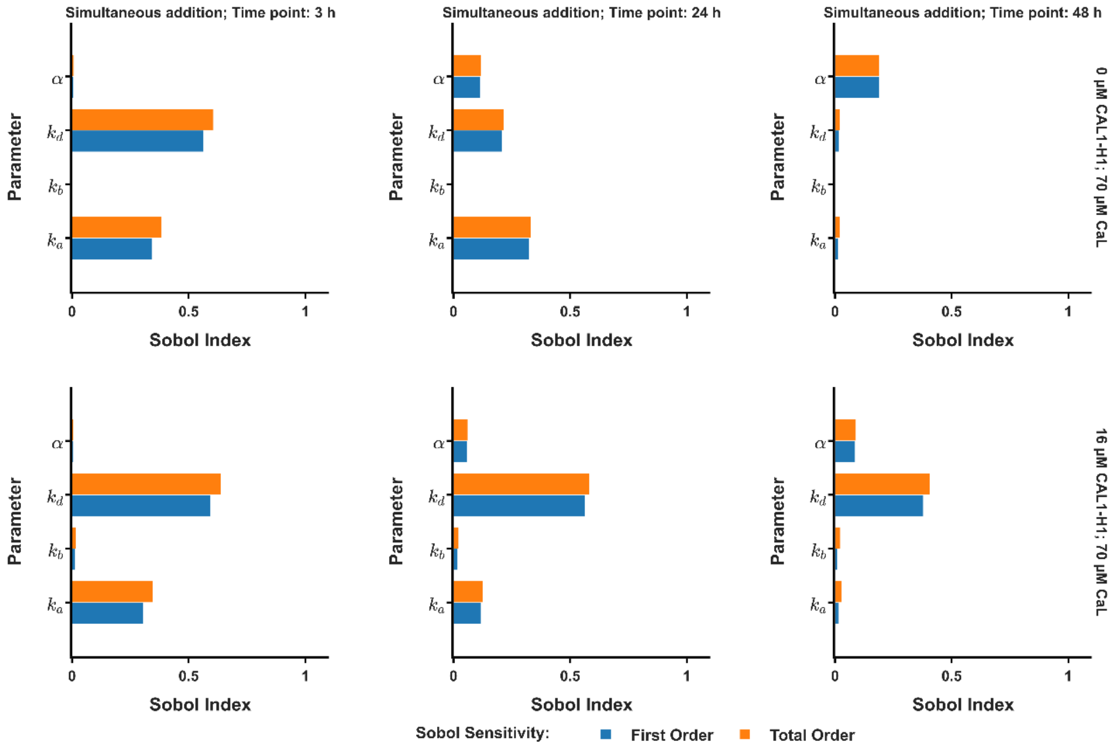

Figure S4. Sobol sensitivity analysis on *in silico* model with synthetic candidalysin. *In silico* model sensitivity after 3 h, 24 h, and 48 h for 70 µM candidalysin (CaL) in the absence and presence of 16 µM anti-candidalysin nanobody (CAL1-H1) that is simultaneously added with candidalysin.

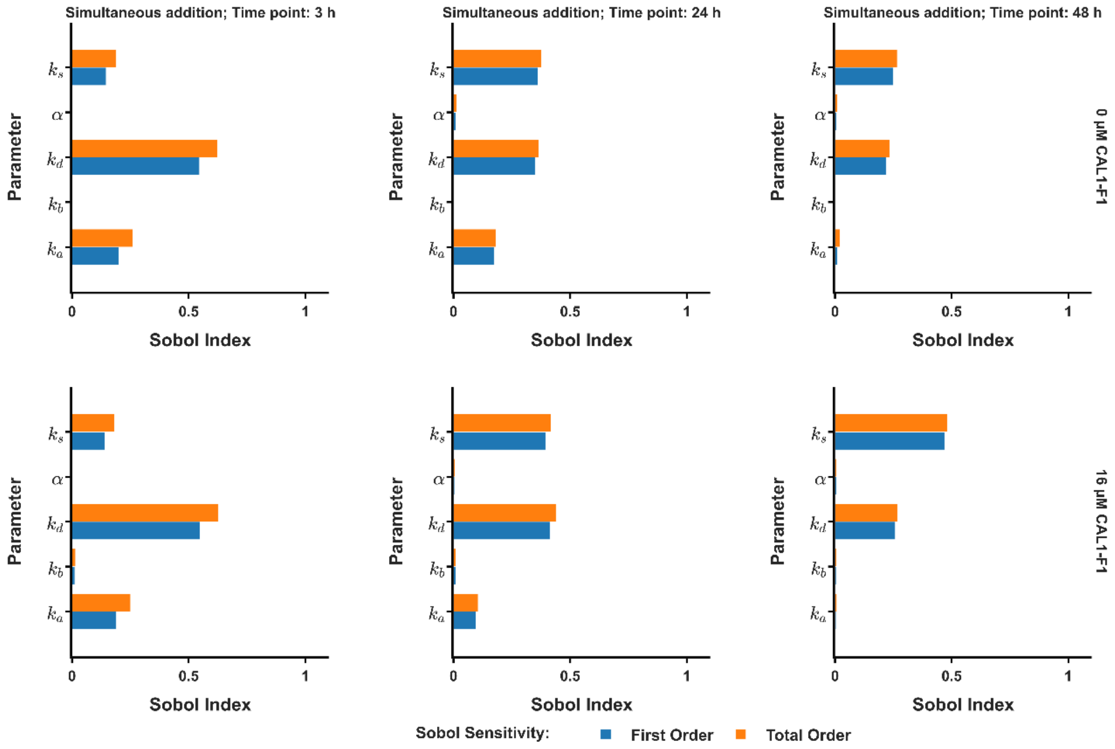

Figure S5. Sobol sensitivity analysis on *in silico* model with native candidalysin. *In silico* model sensitivity after 3 h, 24 h, and 48 h for *C. albicans*-secreted candidalysin (CaL) in the absence and presence of 16 µM anti-candidalysin nanobody (CAL1-F1) that is simultaneously added with *C. albicans*.

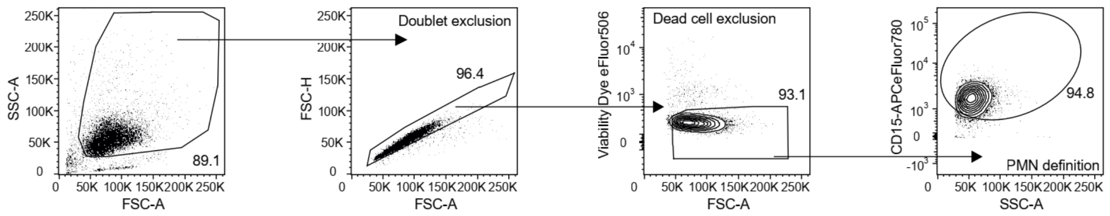

Figure S6. Gating strategy for flow cytometry analysis of neutrophil surface activation markers. Doublet exclusion was followed by dead cell exclusion and out of the viable CD15^{+} neutrophil population mean fluorescence intensities for expression of activation markers were extracted.

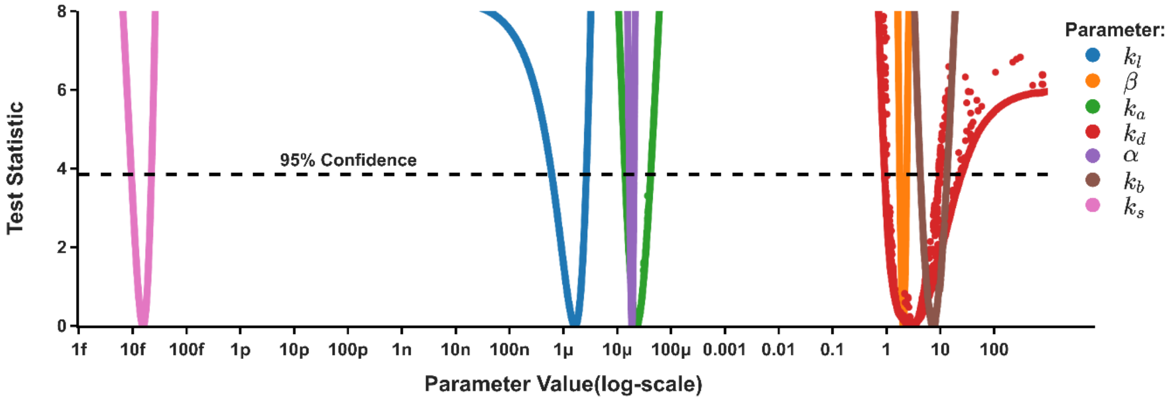

Figure S7. Profile likelihoods of the identifiable parameters. The parameters are depicted in different colors. The dash line depicts the test statistic for a 95% probability of a chi-squred (χ^2) test with one degree of freedom. The y-axis represents the test statistic of χ^2 test with one degree of freedom. Each point represents an optimization run with changed parameter value.

Table S1. *In silico* modelling aggregate size dependency of parameters.

Aggregate Size	α	k_d	k_s
8	1.87e-05	3.44	1.55e-14
16	9.23e-06	6.83	8.69e-15
32	4.67e-06	1.36	4.68e-15
64	2.34e-06	2.74e+01	2.4e-15
128	1.17e-06	5.47e+04	1.22e-15
256	5.82e-07	9.07e+04	6.21e-16
512	2.93e-07	2.17e+04	3.06e-16
1024	1.46e-07	4.23e+04	1.53e-16

Table S2. Antibodies used for western blot.

Antibody	Species	Dilution	Company	Catalogue number
α-actin (clone C4)	Mouse	1:10 000	Merck Millipore	MAB1501
p-DUSP1/MKP1 (S359)	Rabbit	1:1000	Cell Signalling	2857
p-EGFR (Tyr1068)	Rabbit	1:1000	Cell Signalling	2234
c-Fos	Rabbit	1:1000	Cell Signalling	2250
Peroxidase-conjugate AffiniPure anti-mouse IgG	Goat	1:20 000	Jackson ImmunoResearch	115-035-062
Peroxidase-conjugate AffiniPure anti-rabbit IgG	Goat	1:20 000	Jackson ImmunoResearch	115-035-003

Table S3. Primers used for qPCR.

Gene	Forward primer (5'→3')	Reverse primer (5'→3')
ECE1	CACTGGTGTTCAACAATCCAT	AGCATTTTCAATACCGACAG
ACT1	TCAGACCAGCTGATTTAGGTTTG	GTGAACAATGGATGGACCAG
18S rDNA	TAGAGGGACAAGTGGCGTTC	CGCTGAGCCAGTCAGTGT

Table S4. ***In silico*** **modelling parameter and entity description.**

Parameter	Description	Units
A	Aggregate size	1
k_s	Secretion rate for C_M by F_I for effective candidalysin	Ms^{-1}
r	Transition rate from Y to F_{NI}	s^{-1}
r_i	Transition rate from F_{NI} to F_I	s^{-1}
k_n	Degradation rate of Nb	s^{-1}
k_b	Association rate between Nb and C_M or C_A	$s^{-1}M^{-1}$
k_a	Transition rate from C_M to C_A	s^{-1}
k_d	Damaging rate of C_A on E leading to LDH release	$M^{-1}s^{-1}$
k_l	Degradation rate constant of LDH	M^{-1}
α	Conversion constant for usage of aggregate on host cell damage	M
β	Conversion constant for LDH release on host cell death	$ng\ ml^{-1}$
Nb	Concentration of nanobody	M
C_M	Concentration of candidalysin monomers	M
C_A	Concentration of candidalysin aggregates	M
E	Amount of alive VECs	1
LDH	Concentration of lactate dehydrogenase (LDH)	$ng\ ml^{-1}$
F_{NI}	Amount of non-invasive *C. albicans* hyphae	1
F_I	Amount of invasive *C. albicans* hyphae	1
Y	Amount of *C. albicans* yeast cells	1

Note that $[Nb], [C_M], [C_A], [E], [Y], [F_{NI}], [F_I]$, and $[LDH]$ represent the initial concentrations of the corresponding entities at time t=0.
